# Salivary gland transcriptomic analysis and immunophenotyping in the IL-14α transgenic mouse model of Sjögren's disease

**DOI:** 10.3389/fdmed.2025.1612522

**Published:** 2025-07-08

**Authors:** Lucas T. Woods, Kimberly J. Jasmer, Kevin Muñoz Forti, Alex Kearns, Gary A. Weisman

**Affiliations:** ^1^ Department of Biochemistry, University of Missouri, Columbia, MO, United States; ^2^ Christopher S. Bond Life Sciences Center, University of Missouri, Columbia, MO, United States; ^3^ Department of Oral Immunology and Infectious Diseases, University of Louisville School of Dentistry, Louisville, KY, United States; ^4^ Section on Hematology and Oncology, Department of Medicine, The University of Chicago, Chicago, IL, United States

**Keywords:** Sjögren's disease, salivary gland, spatial transcriptome, interleukin-14α transgenic, RNAseq, sialadenitis

## Abstract

Sjögren's disease (SjD) is a systemic autoimmune disorder primarily affecting the exocrine glands and characterized by dry mouth and dry eye, the presence of anti-SSA and/or anti-SSB autoantibodies in blood serum, and chronic lymphocytic infiltration of salivary and lacrimal glands (*i.e.*, sialadenitis and dacryoadenitis, respectively). In addition to reduced quality of life, SjD patients experience severe oral health complications and are at increased risk of developing B cell lymphoma. Because current SjD treatments primarily focus on oral and ocular symptom management, identifying initiating factors and mechanisms of disease progression may offer new therapeutic insights for SjD. The interleukin-14α transgenic (IL-14αTG) mouse model of SjD recapitulates many aspects of human SjD, including progressive sialadenitis, loss of salivary gland function, and development of B cell lymphoma. We utilized immunofluorescence, flow cytometry, bulk RNA sequencing and spatial transcriptomic analyses to identify immune cell subpopulations and differentially expressed genes (DEGs) in submandibular glands of IL-14αTG Sjögren's-like mice and age-matched C57BL/6 mouse controls. We further compared the gene ontology of DEGs in IL-14αTG mice to DEGs identified in minor salivary gland biopsies from SjD patients and healthy volunteers. Results demonstrated significantly increased sialadenitis in IL-14αTG compared to C57BL/6 mice that correlated with an increased proportion of marginal zone B cells infiltrating the submandibular gland. Whole transcriptome analyses showed substantial overlap in enriched DEG ontology between IL-14αTG mouse submandibular gland and SjD patient minor salivary gland, compared to C57BL/6 mice and healthy human volunteer controls, respectively. Lastly, we spatially resolved DEG expression and localization within IL-14αTG salivary glands, marking the first publication of a spatial transcriptomic dataset from submandibular glands in a SjD mouse model.

## Introduction

Sjogren's disease (SjD) is a systemic autoimmune disease characterized by dry eye (*i.e.*, keratoconjunctivitis sicca) and dry mouth (*i.e.*, xerostomia) resulting from chronic lacrimal and salivary gland dysfunction, respectively (1). In addition to increased oral health complications, such as dental caries, candidiasis and periodontitis that degrade quality of life, SjD patients often experience systemic manifestations including pulmonary dysfunction, musculoskeletal pain, fatigue and sleep disturbances (2). Prominent among systemic manifestations is the increased risk of B cell lymphoma in SjD patients, particularly non-Hodgkin mucosa-associated lymphoid tissue (MALT) lymphoma that most often develops in salivary glands, lymph nodes and lung tissues where disease symptoms are active (3). Clinical diagnosis of SjD most commonly occurs in women 40–55 years of age, with an estimated female-to-male ratio of 9:1, and classification criteria have changed over time due to the complexity and systemic nature of SjD (4). In 2016, the American College of Rheumatology (ACR) and the European League Against Rheumatism (EULAR) jointly designed and clinically validated consensus SjD classification methodology for patients with oral or ocular dryness that uses a combination of weighted factors, including the presence of anti-SSA/Ro autoantibodies in blood serum, a positive biopsy showing lymphocytic infiltration of the minor salivary glands (*i.e.*, sialadenitis), and quantitative measurements of ocular staining, saliva flow rate and tear production (1).

Due to the lack of curative therapeutics, current SjD treatments focus on oral and ocular symptom management to improve patient quality of life and systemic anti-inflammatory treatments to limit extra-glandular disease manifestations. Treatments for oral dryness include non-pharmacological interventions such as lozenges, chewing gum, and saliva substitutes and pharmacological interventions such as administration of the muscarinic receptor agonists pilocarpine and cevimeline to stimulate saliva production (5). Treatments for systemic manifestations of SjD include anti-inflammatory and anti-rheumatic drugs such as glucocorticoids, hydroxychloroquine and methotrexate for musculoskeletal pain and respiratory symptoms. However, data regarding clinical efficacy of these treatments is limited (6). Because SjD patients exhibit many hallmarks of B cell hyperactivity, including the development of anti-SSA/Ro autoantibodies, the presence of ectopic germinal centers in the salivary glands, and increased risk of B cell lymphoma (5, 7, 8), B cell-targeted therapies have also been utilized to treat systemic symptoms of SjD. In clinical trials, the anti-CD20 monoclonal antibody rituximab is the most heavily studied B cell-targeted therapy (9–11) along with the anti-CD22 monoclonal antibody epratuzumab (12), the anti-B lymphocyte stimulator (BlyS; BAFF) monoclonal antibody belimumab (13) and the small molecule inhibitor remibrutinib that targets Bruton's tyrosine kinase (14). However, due to the lack of consensus data on the efficacy of B cell-targeted therapies in SjD, EULAR recommendations limit their use to patients with severe refractory SjD after failure of conventional therapies (5). Considering the limited treatment options for symptom management and the lack of effective treatments for underlying disease processes, identification of disease-initiating factors and mechanisms of progression may offer new therapeutic insights for SjD treatment.

Investigating SjD progression in human patients presents difficulties resulting from unknown disease etiology and the potential delay between disease onset and clinical diagnosis. Therefore, numerous SjD animal models have been developed to study disease progression, including spontaneous models such as non-obese diabetic (NOD) mouse derivatives, knockout models including *CD25*^−/−^ and *Id3*^−/−^ mice, transgenic models such as the BlyS/BAFF transgenic mouse, and induced models following mouse immunization with muscarinic receptor 3 or SSA/Ro antigens (15). The interleukin-14α transgenic (IL-14αTG) mouse model of SjD was generated by over-expressing human interleukin-14 (alpha-taxilin; high molecular weight B cell growth factor) under the direction of the lymphoid *IgH* promoter in the C57BL/6 mouse background (16). IL-14αTG mice exhibit many aspects of B cell hyperactivation, including hypergammaglobulinemia, increased immunoglobulin production in response to T cell-dependent and -independent antigens, and increased levels of B cell subpopulations in the spleen and peritoneum (16). These mice also recapitulate many aspects of human SjD, including progressive sialadenitis, loss of salivary gland function and development of B cell lymphoma (16–19).

The widespread application of whole transcriptome analysis through bulk and single-cell RNA sequencing (RNAseq) has greatly advanced our understanding of molecular pathways involved in SjD pathogenesis, highlighting previously unknown gene expression changes and cellular heterogeneity in SjD salivary gland biopsies (20–24). Similarly, transcriptomic analyses of mouse salivary glands during development have elucidated the heterogeneity of salivary gland parenchyma from embryonic stage through adulthood (25). Other studies in SjD mouse models have identified previously unknown salivary and lacrimal gland signaling paradigms that are altered during SjD progression, including the downregulation of metabolic pathways involved in amino acid metabolism and fatty acid biosynthesis, the enrichment of diverse innate and humoral inflammatory pathways and the contributions of salivary epithelial cells to chronic immune dysregulation (26, 27). Unlike bulk and single-cell RNAseq, spatial transcriptomic analysis allows gene transcripts to be identified with their spatial context still intact and provides additional indications of cellular interactions within the tissue (28). By overlaying histological tissue sections on an array of barcoded oligo(dT) primers or pre-designed capture probes followed by RNAseq, mRNA transcripts can be quantified and localized without the limitation of dissociating individual intact cells from whole tissue that can stress or damage labile cells such as neurons (29). Spatial transcriptomics analysis has been previously utilized to probe epithelial-immune cell interactions and stratify acinar, ductal and T cell subsets within tissue niches of SjD minor salivary glands (30, 31) and, alongside bulk RNAseq, to identify tissue localization of differentially expressed genes (DEGs) and cell cluster-specific gene pathway enrichment in inflamed lacrimal glands of the NOD.B10-H2^b^ mouse model of SjD (26).

Here, we utilized immunofluorescence, flow cytometry and bulk RNAseq analyses to compare immune cell subpopulations and differentially expressed genes in IL-14αTG and age-matched control C57BL/6 mouse submandibular glands (SMGs). We further compared the gene ontology of DEGs in IL-14αTG mice to DEGs identified in minor salivary gland biopsies from SjD patients and healthy volunteers. Lastly, we utilized spatial transcriptomic analyses to localize the expression of DEGs within the SMG and sublingual glands of IL-14αTG and C57BL/6 mice. To our knowledge, this is the first presentation of spatial transcriptomic data from submandibular and sublingual glands of an SjD mouse model.

## Materials and methods

### Mice

C57BL/6 (stock # 000664) mice were purchased from Jackson Laboratories (Bar Harbor, ME) and IL-14αTG mice were housed at the Christopher S. Bond Life Sciences Center Animal Facility of the University of Missouri (Columbia, MO). Animals were housed in vented cages with 12 h light/dark cycles and received food and water *ad libitum*. Age-matched female mice were used in all experiments and genotyping was performed by PCR, as previously described (16). Euthanasia was performed by terminal anesthesia with isoflurane followed by cervical dislocation, with efforts taken to minimize suffering. All experimental animal procedures were conducted in accordance with National Institutes of Health guidelines that were approved by the University of Missouri Animal Care and Use Committee (Protocol Number 38921).

### Bright field and immunofluorescence microscopy

For bright field microscopy, mouse SMGs and sublingual glands (SLGs) were excised, fixed in 4% (v/v) paraformaldehyde for 24 h and dehydrated by incubation in 70% (v/v) ethanol for 24 h at 4°C. Samples were sent to IDEXX BioAnalytics (Columbia, MO), where they were embedded in paraffin, sectioned and stained with hematoxylin and eosin. Stitched images of whole submandibular and sublingual glands were captured on a Leica DMI6000B inverted microscope using LAS X software.

For immunofluorescence analysis, SMGs and SLGs were excised, embedded in OCT compound, snap frozen in 2-methylbutane cooled with liquid nitrogen and cryosectioned on a Leica CM3050 cryostat at the University of Missouri Advanced Light Microscopy Core (UMALMC) Facility. Cryosections were adhered to slides and then fixed in 4% (v/v) paraformaldehyde for 20 min at room temperature or in acetone at −20°C for 10 min (for detection of GL7), washed in PBS and placed for 1 h at room temperature in blocking buffer containing 5% (v/v) goat serum in PBS with mouse BD Fc Block (1:250, BD Biosciences). Sections were then stained for 16 h at 4°C in blocking buffer containing the following conjugated primary antibodies: AlexaFluor 594 rat anti-B220 (1:200, Biolegend clone RA3-6B2), AlexaFluor 488 hamster anti-CD3 (1:200, ThermoFisher clone 145-2C11), Brilliant Violet 421 rat anti-CD169/Siglec-1 (1:200, Biolegend clone 3D6.112), AlexaFluor 594 hamster anti-CD11c (1:200, Biolegend clone N418) or FITC rat anti-GL7 (1:250, BD Biosciences clone GL7). For unconjugated primary antibodies, sections were stained for 16 h at 4°C in blocking buffer containing rat anti-CD45 (1:100, Biolegend clone 30-F11) and rabbit anti-Aquaporin 5 (1:100, MilliporeSigma #178615) antibodies, washed in PBS, then stained with AlexaFluor 594 goat anti-rat IgG (1:1,000, ThermoFisher #A-11007) and AlexaFluor 488 goat anti-rabbit IgG (1:1,000, ThermoFisher #A-11008) secondary antibodies for 1 h at room temperature. Slides were then washed in PBS and coverslips mounted using Fluoroshield with DAPI (MilliporeSigma). Stitched and high-magnification SMG and SLG images were captured on a Leica STELLARIS 5 confocal microscope using LAS X software.

### Flow cytometry

SMGs were isolated, finely minced in digestion media [RPMI-1640 media containing 5% (v/v) fetal bovine serum, 2 mM EDTA, 2.5 mM CaCl_2_ and 1 mg/ml collagenase D (MilliporeSigma)], and placed in a shaking incubator for 2 h at 37°C and 270 rpm. Dispersed SMGs and isolated spleens were then passed through a 40 µm cell strainer, washed in PBS and spleen homogenates were resuspended in red blood cell lysis buffer (Miltenyi Biotec) for 10 min in the dark. Following a wash in PBS, cells were pelleted by centrifugation at 500×g at 4°C, resuspended in PBS, counted and aliquoted for analysis.

For antibody staining, 10^6^ cells were resuspended in 100 µl of PBS containing Zombie NIR viability dye (1:2,000) and mouse BD Fc Block (1:100), incubated for 15 min in the dark, and then washed in cytometry buffer [0.5% (w/v) bovine serum albumin, 2 mM EDTA in PBS]. Cells were then resuspended in cytometry buffer containing 1:100 dilutions of each antibody in either the B cell or T cell panel (Table 1) and incubated for 1 h at 4°C in the dark. Following a 5 min wash in cytometry buffer, cells were fixed and permeabilized using the eBioscience FoxP3/transcription factor staining buffer set (ThermoFisher) per the manufacturer's protocol. Cells were then resuspended in eBioscience permeabilization buffer containing 1:100 dilutions of antibodies targeting intracellular antigens (denoted with # in Table 1) and incubated for 16 h at 4°C in the dark. Cells were then washed twice and resuspended in cytometry buffer for analysis, with an aliquot of unstained sample reserved for autofluorescence control. Fluorescence minus one (FMO) control samples and single-stained compensation controls were similarly prepared using spleen cells for FMOs and UltraComp eBeads Plus compensation beads (ThermoFisher), respectively. Samples were analyzed on a Cytek Aurora spectral flow cytometer at the University of Missouri Cell and Immunobiology Core Facility and spectral unmixing was performed using Cytek SpectroFlo software. Representative gating strategies for each panel are shown in Supplementary Figures S1 and S2.

**Table 1:** Flow cytometry antibodies used for identification of B and T cell subpopulations, where # denotes an intracellular antigen.

FACS panel	Antigen	Fluorophore	Clone	Catalog number
B and T cells	Viability dye	Zombie NIR	–	Biolegend 423106
	CD45	VioBlue	REA737	Miltenyi 130-110-664
T cells	CD3ε	PerCP/Cyanine 5.5	145-2C11	Biolegend 100328
	CD4	Brilliant Violet 750	GK1.5	Biolegend 100467
	CD8a	PE/Dazzle 594	53-6.7	Biolegend 100762
	CD69	PE/Cyanine 7	H1.2F3	Biolegend 104512
	CD279	PE	HA2-7B1	Miltenyi 130-102-299
	FoxP3#	APC	FJK-16s	Invitrogen 17-5773-82
	CD25	APC/Cyanine 7	3C7	Biolegend 101918
	GATA3#	AlexaFluor 488	16E10A23	Biolegend 653808
	RORγt#	PerCP/eFluor 710	B2D	Invitrogen 46-9681-82
	CD183	Super Bright 600	CXCR3-173	Invitrogen 63-1831-82
B cells	CD19	PerCP	6D5	Biolegend 115532
	B220 (CD45R)	APC	REA755	Miltenyi 130-110-710
	CD5	PerCP/Cyanine5.5	53-7.3	Biolegend 100624
	CD23	PE/CF594	B3B4	BD Biosciences 563986
	CD21/CD35	PE	7E9	Biolegend 123410
	GL7	AlexaFluor 488	GL-7	Invitrogen 53-5902-82
	Bcl-6#	BV421	K112-91	BD Biosciences 563363
	CD38	PerCP/eFluor 710	90	Invitrogen 46-0381-82
	CD273	BV786	TY25	BD Biosciences 741026
	CD138	APC/Cyanine7	281-2	Biolegend 142530

### Bulk RNA sequencing and gene ontology analysis

SMGs from 6- and 12-month-old IL-14αTG and C57BL/6 mice were excised, SLGs were removed, and SMG tissue was homogenized in 1 ml TRIzol reagent (ThermoFisher) using a handheld homogenizer. Next, 0.2 ml of chloroform was added, and samples were vortexed for 20 s and allowed to sit at room temperature for 15 min. Samples were centrifuged at 10,000×g for 18 min at 4°C and the resulting aqueous phase was passed through a genomic DNA Eliminator column from the RNeasy Plus Mini kit (Qiagen) before RNA isolation was performed per the manufacturer's protocol. Purified RNA concentration was calculated using a NanoDrop One spectrophotometer and 1 µg of RNA was used for sequencing library preparation using the Stranded mRNA Prep kit (Illumina) at the University of Missouri Genomics Technology Core (UMGTC) Facility. Total mRNA integrity was assessed on an Agilent 5200 Fragment Analyzer before whole transcriptome sequencing to a depth of at least 50 million reads per sample was performed on a NovaSeq 6000 sequencing system (Illumina).

Raw FASTQ data from mouse SMGs (*n* = 3/genotype/timepoint) and from human minor salivary gland biopsies of SjD patients and healthy volunteers [*n* = 9 healthy volunteers and 35 SjD patients; accessed through the National Center for Biotechnology Information's Database of Genotypes and Phenotypes (dbGAP) accession phs001842.v1.p1] (20) were processed by the University of Missouri Bioinformatics and Analytics Core (UMBAC) Facility. Sequence read quality was assessed using FastQC and reads were filtered and trimmed using fastp (32), then mapped against the *Mus musculus* reference genome GRCm39 or the *Homo sapiens* reference genome GRCh38 using STAR software (33). Data were normalized by variance stabilizing transformation using the DESeq2 R package (34) and sample variance was assessed by principal component analysis (PCA). Differential gene expression analysis was performed using DESeq2, where differentially expressed genes were defined as those having a greater than 2-fold change (Log2FC ≥ 1) and adjusted *P* value less than 0.05 [-Log10(padj) ≥ 1.3]. Volcano plots of DEGs were generated using VolcaNoseR (35) and DEG ontology, Kyoto Encyclopedia of Genes and Genomes (KEGG) pathway/biological process enrichment, and functional annotation analysis was performed using the Database for Annotation, Visualization, and Integrated Discovery (DAVID) (36, 37).

### Spatial transcriptomic analysis

Spatial transcriptomic analysis was carried out using the poly-(dT) capture primer-based Visium v1 spatial gene expression assay from 10X Genomics (Pleasanton, CA) according to the manufacturer's protocols. Briefly, SMGs and SLGs were excised from 12-month-old IL-14αTG and C57BL/6 mice, embedded in OCT compound, snap frozen in 2-methylbutane cooled with liquid nitrogen, and cryosectioned to 10 µm thickness on a Leica CM3050 cryostat. For tissue optimization, cryosections were adhered to a Visium spatial tissue optimization slide and the optimum SMG and SLG tissue permeabilization time was determined to be 18 min. For spatial gene expression analysis, SMG and SLG cryosections were adhered to a Visium spatial gene expression slide, stained with hematoxylin and eosin, and imaged on a Zeiss Axiovert 200M inverted microscope at the UMALMC Facility. Serial tissue cryosections were also collected for immunofluorescence analysis of spatial gene expression tissues. Next, mRNA libraries were prepared and sequenced to a depth of 50,000 reads/spot on an Illumina NovaSeq 6000 sequencing system at the UMGTC Facility and FASTQ files were processed using the Space Ranger 2.0.0 pipeline at the UMBAC Facility. Spatial gene expression visualization was carried out using Loupe Browser 8.1 (10X Genomics). Unsupervised Louvain clustering using PCA embedding and a resolution of 1 was performed using BioTuring Lens, and cell cluster annotation was performed using BioTuring Talk2Data and SpatialX modules.

### Statistical analysis

Flow cytometry data were analyzed using GraphPad Prism version 10.2.0. Data were first assessed for normality using Shapiro–Wilk test and data with normal distribution were analyzed by one-way ANOVA followed by Sidak correction for multiple comparisons or unpaired two-tailed t test. Data that did not fit normal distribution were analyzed by Kruskal–Wallis test followed by Dunn's test for multiple comparisons or Mann–Whitney test.

### Data availability

The flow cytometry data presented in this study were deposited at ImmPort Shared Data database (accession SDY3118). Raw and normalized bulk RNAseq data from IL-14αTG and C57BL/6 mice are provided in Supplementary Table S2. Bulk RNAseq data from human SjD patients and healthy volunteers are available from dbGAP (accession phs001842.v1.p1). Spatial transcriptomic datasets were deposited at the National Center for Biotechnology Information's Gene Expression Omnibus (accession GSE298921).

## Results

### Progressive sialadenitis in Il-14αTG mice

**Figure 1:**
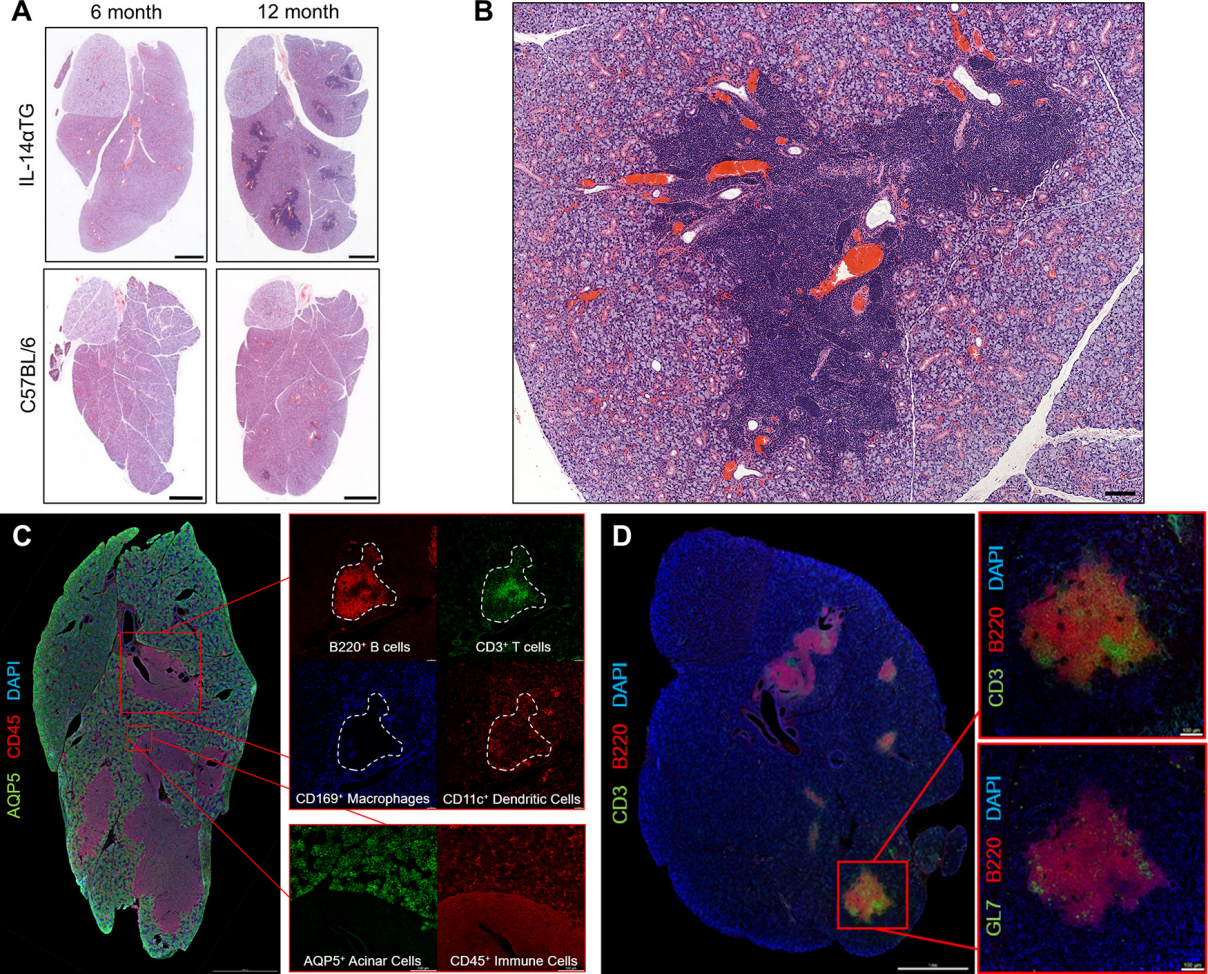
Progressive sialadenitis in the IL-14αTG mouse model of Sjögren's disease. **(A)** Submandibular and sublingual glands from 6- and a 12-month-old female IL-14αTG and C57BL/6 mice were subjected to hematoxylin and eosin staining to assess glandular inflammation; scale bar = 1 mm. **(B)** Immune cell focus surrounding blood vessels and excretory ducts in a 12-month-old IL-14αTG mouse SMG; scale bar = 100 µm. **(C)** Twelve-month-old and **(D)** 18-month-old female IL-14αTG mouse SMG and SLG cryosections were subjected to immunofluorescence staining using antibodies against aquaporin 5 (AQP5) acinar cell marker, CD45 pan-immune cell marker, B220 B cell marker, CD3 T cell marker, CD169 macrophage marker, CD11c dendritic cell marker or GL7 germinal center marker with DAPI nuclear counterstain; scale bars = 1 mm and 100 µm (inset).

From 6 to 18 months of age, IL-14αTG mice develop progressive inflammation of the submandibular and parotid glands while the sublingual glands are less affected (18). Hematoxylin and eosin staining of 6- and 12-month-old IL-14αTG mouse salivary glands revealed the presence of numerous large inflammatory foci in the SMG at 12 months of age, as compared to age-matched C57BL/6 control mouse SMG, but not in the SLG (Figure 1A). These inflammatory foci were each positioned around a central blood vessel and large excretory duct (Figure 1B) and stained positive for the pan-immune cell marker CD45, whereas aquaporin 5 (AQP5)^+^ salivary acinar cells were only present in the surrounding SMG tissue (Figure 1C). Immune cell type-selective markers identified B220^+^ B cells and CD3^+^ T cells that occupied distinct areas within each focus (Figure 1C, inset). CD11c^+^ dendritic cells were present throughout the focus and the surrounding SMG tissue, while CD169^+^ macrophages were present at the focus perimeter and throughout the surrounding SMG tissue (Figure 1C, inset). Some lymphoid structures in the IL-14αTG SMG also stained positive for the activated B and T cell antigen GL7 (Figure 1D), a marker for activated B and T cells present in germinal centers (GC) where B cells undergo antibody affinity maturation and clonal expansion (38, 39).

**Figure 2:**
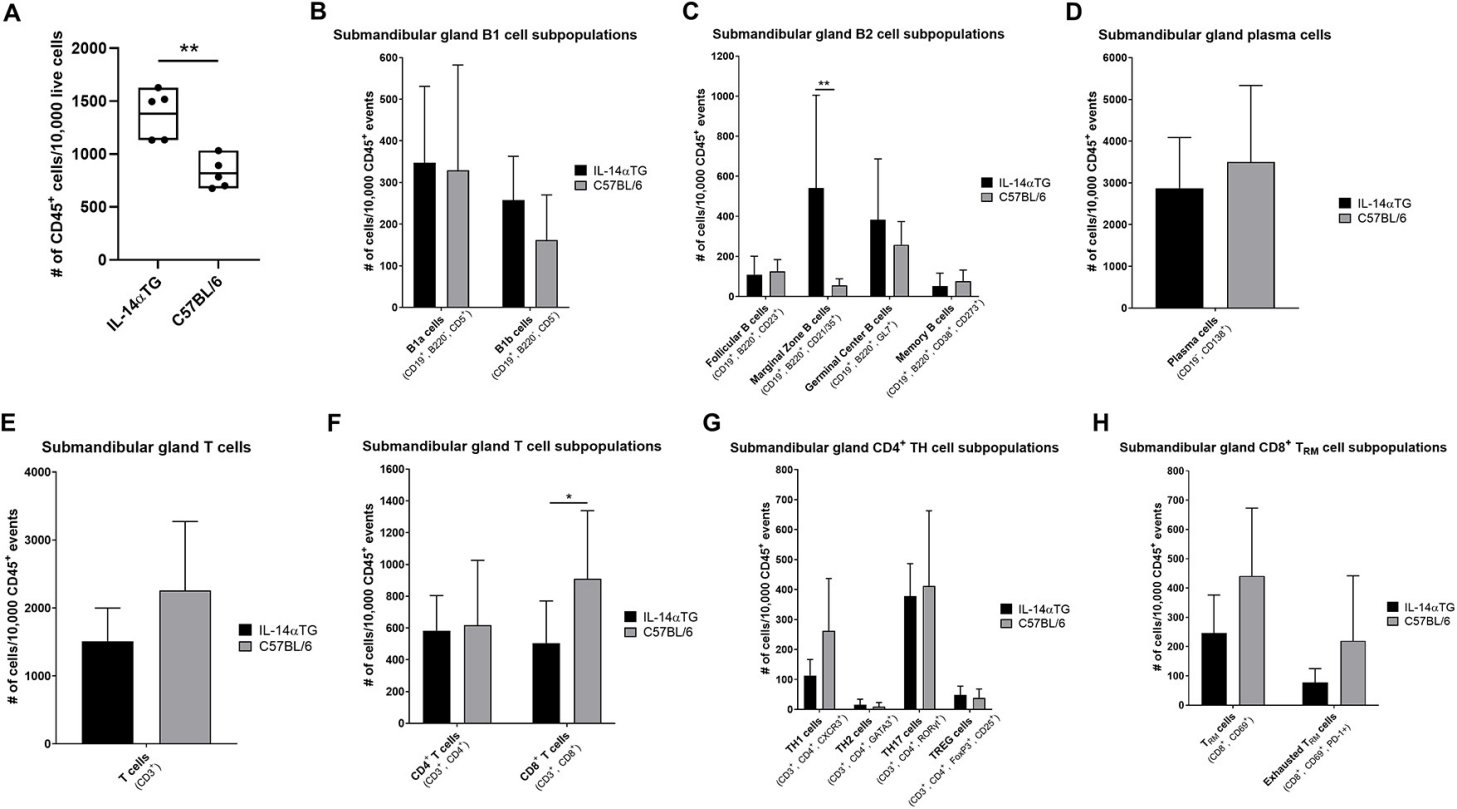
Flow cytometry analysis of IL-14αTG and C57BL/6 mouse SMG-infiltrating immune cells. SMGs from 12-month-old IL-14αTG and C57BL/6 mice were enzymatically dispersed, stained with either a B or T cell type-specific antibody panel and analyzed by flow cytometry on a Cytek Aurora spectral analyzer. **(A)** Total CD45^+^ immune cells, **(B–D)** B cell subpopulations and **(E–H)** T cell subpopulations were quantified, and data are presented as means ± S.D., where * and ** indicate *P* < 0.05 and 0.01, respectively, for *n* = 5 mice per group.

Because the sublingual glands lacked immune cell foci and are the least affected salivary glands in SjD patients (40), flow cytometry was performed on immune cells isolated from SMGs at the 12-month time point and the results reflected histological findings, where a significant increase in CD45^+^ immune cells was observed in IL-14αTG mice as compared to age-matched C57BL/6 mice (Figure 2A). Previous studies identified alterations in innate B1 and conventional B2 cell subpopulations in IL-14αTG vs. littermate control mice (16); therefore, we compared B lymphocyte subpopulations and initially stratified cells based on CD19, B220 and CD5 expression. B1 cells, which have long been implicated in the development of autoimmunity and production of autoantibodies (41), were identified in both IL-14αTG and C57BL/6 mouse SMGs; however, no significant differences were observed between genotypes with regards to either B1a (CD19^+^, B220^−^, CD5^+^) or B1b (CD19^+^, B220^−^, CD5^−^) cell subsets (Figure 2B). In the conventional B2 cell compartment (CD19^+^, B220^+^), cell fate decisions determining differentiation into recirculating short-lived follicular B cells (CD23^+^) or long-lived static marginal zone (MZ) B cells (CD21/35^+^) are dictated by the strength of B cell receptor (BCR) signaling (42), and both follicular and MZ B cells were identified in IL-14αTG and C57BL/6 mouse SMGs (Figure 2C). Follicular B cells represented ∼1% of total immune cells in both genotypes and levels were not significantly different; however, the proportion of MZ B cells was significantly increased 5-fold in IL-14αTG mouse SMG as compared to controls (Figure 2C). GC B cells (GL7^+^) were present in the IL-14αTG mouse SMG, confirming observations of GL7 immunofluorescence staining (Figure 1D), and we observed similar levels of GL7^+^ B cells in C57BL/6 mouse control SMGs (Figure 2C). In both genotypes, long-lived memory B cells (CD38^+^, CD273^+^) represented the least abundant B cell population (∼0.6%; Figure 2C) and antibody producing plasma cells (CD19^−^, CD138^+^) were the most abundant population, representing 30%–35% of all CD45^+^ SMG-infiltrating immune cells (Figure 2D). However, no statistical differences between genotypes were observed in either population.

We next examined T cell subpopulations in the SMG and found no significant differences in the proportion of total CD3^+^ T cells (Figure 2E) or CD4^+^ T helper (TH) cells (Figure 2F) between IL-14αTG and C57BL/6 mice. Further analysis of CD4^+^ TH subsets, including TH1 (CXCR3^+^), TH2 (GATA3^+^), TH17 (RORγt^+^) and TREG (FoxP3^+^, CD25^+^) cells, did not reveal significant differences between IL-14αTG and C57BL/6 mouse SMG (Figure 2G). In both genotypes, TH17 cells made up the highest proportion of total CD45^+^ immune cells among all TH cell subsets (∼4%), whereas TH2 cells made up the lowest proportion (< 0.1%). Additionally, all SMG-infiltrating TREG cells in both genotypes were found to express the activation marker CD69 that enhances the immunomodulatory capacity of these cells (Supplementary Figure S3) (43). In contrast to the CD4^+^ TH cell populations, we observed a significant increase in the proportion of CD8^+^ T cells in C57BL/6 as compared to IL-14αTG mouse SMG (Figure 2F). Within the CD8^+^ T cell compartment, we further analyzed the proportion and activation state of the non-recirculating long-lived T resident memory (TRM) cells (44) using the TRM cell marker CD69 and the exhausted T cell marker programmed death-1 (PD-1), respectively, but found no significant differences in TRM levels nor exhaustion state between genotypes (Figure 2H).

### Common and unique transcriptomic changes in Il-14αTG mouse and SjD patient salivary glands

**Figure 3:**
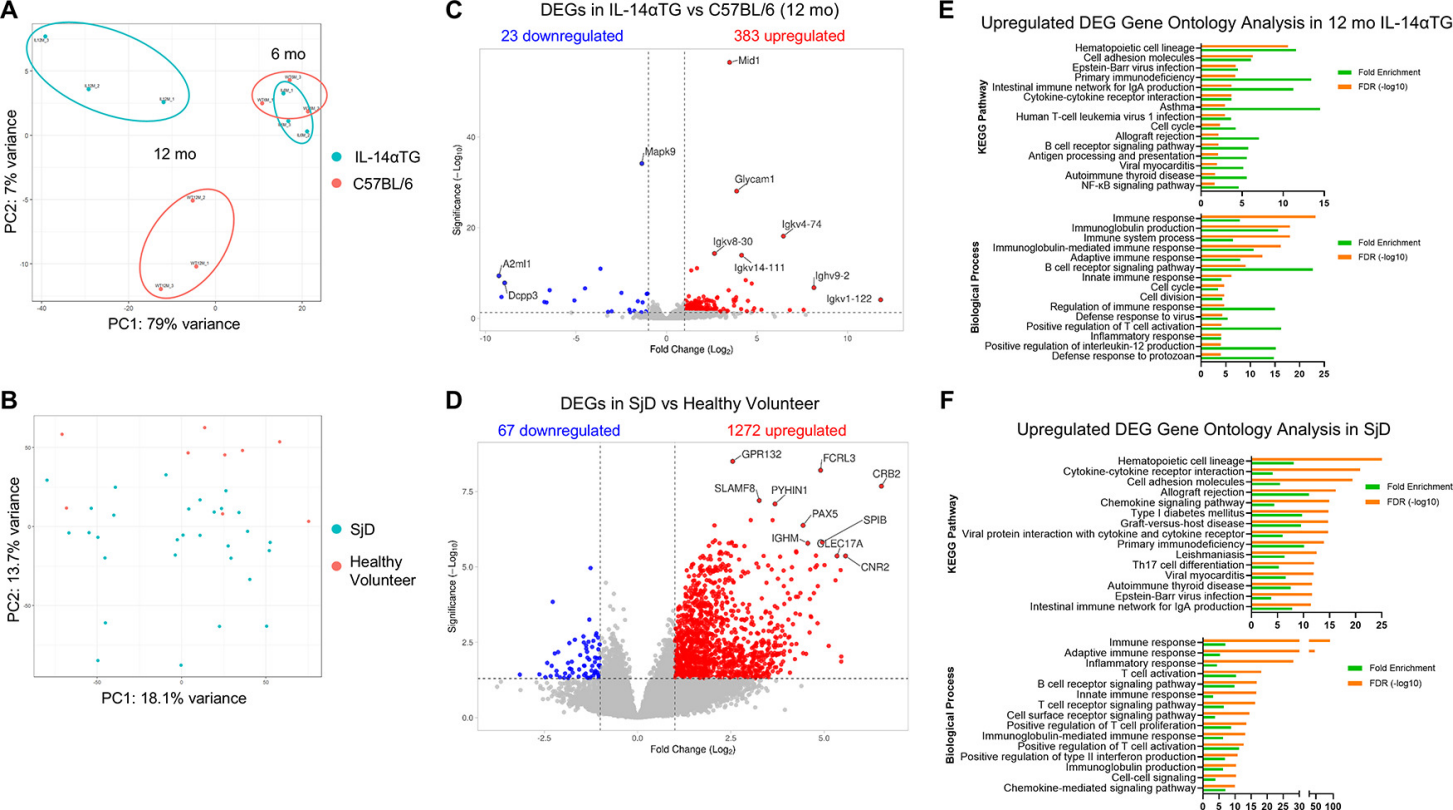
Comparison of differentially expressed genes from IL-14αTG mouse submandibular glands and human SjD minor salivary glands. RNA prepared from 6- and 12-month-old IL-14αTG and C57BL/6 SMGs was subjected to RNAseq analysis. Previously performed RNAseq analysis of SjD patient and healthy volunteer MSG was accessed through dbGAP accession phs001842.v1.p1. Up- and downregulated DEGs were identified and analyzed by **(A,B)** principal component analysis, **(C,D)** DEG volcano plots and **(E,F)** DAVID gene ontology analysis, where **(A,C,E)** are results from IL-14αTG vs. C57BL/6 mouse SMGs (*n* = 3/timepoint/genotype) and **(B,D,F)** are results from SjD patient vs. healthy volunteer MSG biopsies (*n* = 35 SjD patients and 9 healthy volunteers).

Bulk RNAseq analysis of SMGs from 6-month-old and 12-month-old IL-14αTG and C57BL/6 mice identified upregulated and downregulated DEGs in multiple transcriptome comparisons. In the mouse SMG, dramatic changes in the transcriptomic landscape occurred during aging, where the 12-month-old vs. 6-month-old IL-14αTG mouse SMG comparison identified 1,653 total DEGs and 12-month-old vs. 6-month-old C57BL/6 mouse SMG comparison identified 508 DEGs (Table 2; Supplementary Figure S4). Comparison of age-matched IL-14αTG and C57BL/6 mouse SMGs at the 6-month and 12-month timepoints yielded 25 DEGs and 406 DEGs, respectively. We further analyzed human DEGs from bulk RNAseq analysis of minor salivary gland (MSG) biopsies from SjD patients and healthy volunteers (accessed through dbGAP accession phs001842.v1.p1) (20) and found 1,339 DEGs (Table 2). Principal component analysis of mouse SMG RNAseq data showed that IL-14αTG and C57BL/6 mouse SMGs grouped tightly at the 6-month time point, but diverged at the 12-month time point, with PC1 accounting for 79% of variance between samples compared to 7% of the variance for PC2 (Figure 3A). PCA of human minor salivary gland samples demonstrated a similar magnitude of variance across both PC1 and PC2 axes and diffuse grouping (Figure 3B). Volcano plots of DEGs in 12-month-old IL-14αTG vs. C57BL/6 mouse SMGs (Figure 3C) and SjD patient vs. healthy volunteer (HV) MSGs (Figure 3D) show the significance (-Log_10_ padj) and magnitude (Log_2_-fold change) of up- and downregulated DEGs, with annotated dots denoting the top 10 DEGs based on Manhattan distance from origin. Gene ontology analysis of all upregulated DEGs revealed a significant enrichment of KEGG pathways and biological processes associated with immune responses, autoimmunity, and viral infection with substantial overlap noted between IL-14αTG SMG (Figure 3E) and SjD patient MSG biopsies (Figure 3F). In total, 66 biological processes and 22 KEGG pathways were significantly enriched [False Discovery Rate (FDR) < 0.05] in mouse SMG and 169 biological processes (BP) and 68 KEGG pathways were significantly enriched in human MSG (Supplementary Table S1). Gene ontology analysis of downregulated DEGs did not identify any significantly enriched pathways or processes. Interestingly, gene ontology analysis of DEGs associated with aging (*i.e.*, 12-month vs. 6-month) in either C57BL/6 or IL-14αTG mouse genotypes showed enrichment of inflammatory KEGG pathways and biological processes that mirrored those seen in diseased vs. control samples (Supplementary Table S1), corroborating recent reports on the presence of chronic inflammatory pathologies in aged C57BL/6 mouse SMG and lacrimal glands (38, 45).

**Table 2:** Differentially expressed genes identified in whole tissue RNAseq analysis of 6-month-old and 12-month-old IL-14αTG and C57BL/6 mouse SMGs and minor salivary gland biopsies from Sjögren's disease patients and healthy volunteers.

Transcriptome comparison	Upregulated DEGs	Downregulated DEGs
12 mo C57BL/6 vs. 6 mo C57BL/6	503	5
12 mo IL-14αTG vs. 6 mo IL-14αTG	1,627	26
12 mo and 6 mo IL-14αTG vs. 12 mo and 6 mo C57BL/6	10	12
6 mo IL-14αTG vs. C57BL/6	14	11
12 mo IL-14αTG vs. C57BL/6	383	23
Sjögren's disease vs. healthy volunteers	1,272	67

**Figure 4:**
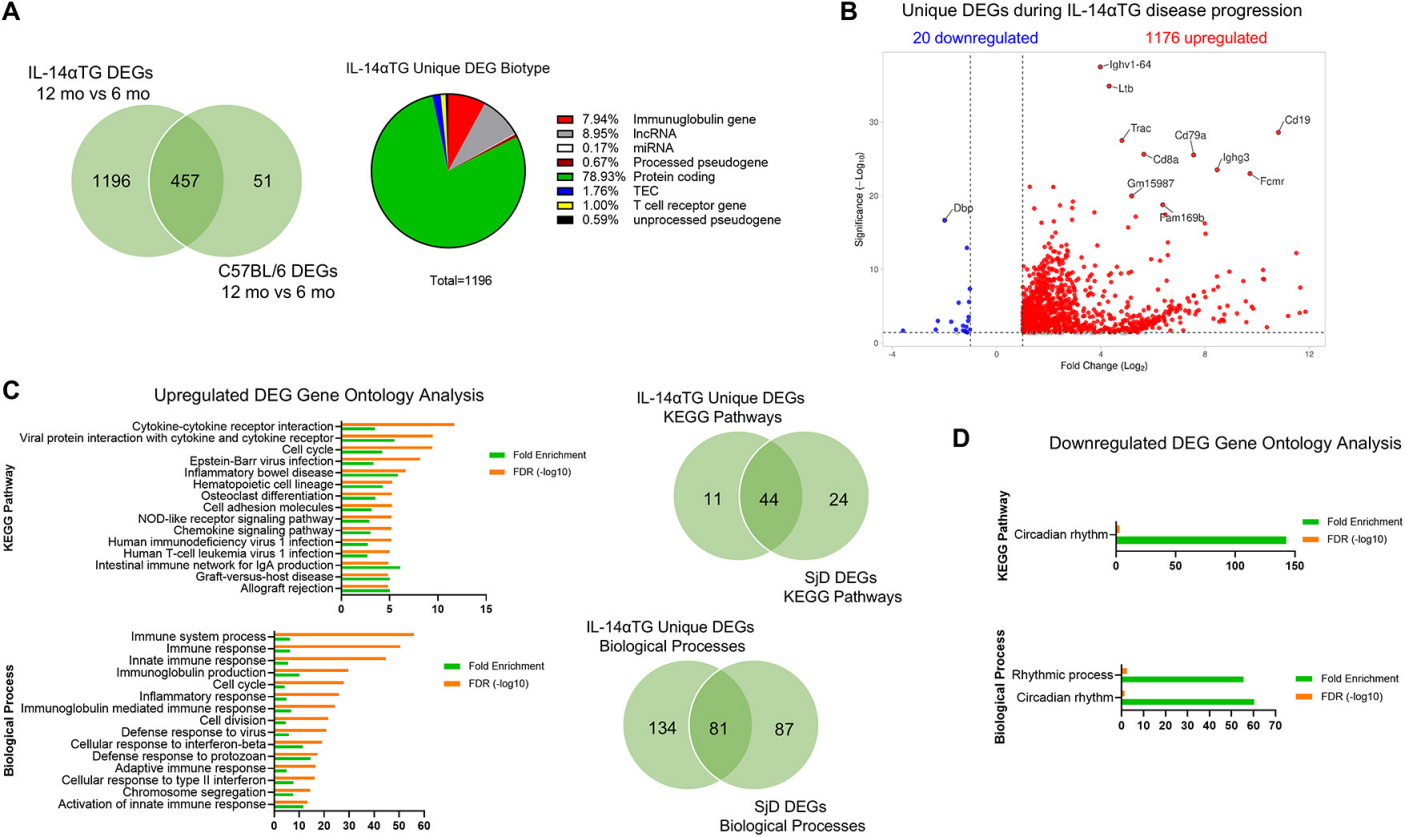
Unique differentially expressed genes in IL-14αTG mouse submandibular glands. DEG lists from 12-month-old vs. 6-month-old IL-14αTG and 12-month-old vs. 6-month-old C57BL/6 mouse SMGs were compared and genes that were differentially expressed with age in both genotypes were removed to generate a list of DEGs unique to IL-14αTG SMG. **(A)** Venn diagram of shared and unique genes differentially expressed with age and pie chart of IL-14αTG unique DEG biotype; TEC,To be Experimentally Confirmed. **(B)** Volcano plot of unique DEGs in IL-14αTG mouse SMG. **(C)** DAVID gene ontology analysis of upregulated unique IL-14αTG mouse SMG DEGs to identify enriched KEGG pathways and biological processes, with Venn diagrams denoting shared and unique signaling pathway enrichment between IL-14αTG mouse SMG and human SjD MSG biopsies. **(D)** DAVID gene ontology analysis of downregulated unique IL-14αTG mouse SMG DEGs.

Next, we compared DEGs in 12-month-old vs. 6-month-old IL-14αTG mouse SMGs and 12-month-old vs. 6-month-old C57BL/6 mouse SMGs and removed genes that were differentially expressed in both genotypes, reasoning that these genes would be associated with aging and the unique IL-14αTG mouse DEGs would be disease model-specific. In total, 457 DEGs were common between both genotypes, leaving 1,196 DEGs unique to the IL-14αTG mouse SMG disease model (Supplementary Table S1). When grouped by gene biotype, protein coding genes made up the majority of IL-14αTG mouse SMG unique DEGs (∼79%) followed by long non-coding RNA (lncRNA) and immunoglobulin genes (Figure 4A). The top 10 unique IL-14αTG DEGs based on Manhattan distance were identified by volcano plot and included the IgH constant and variable chain genes *Ighg3* and *Ighv1-64* (homologous to human *Ighv1-24*), the B cell genes *Cd19* and *Cd79a*, the T cell genes *Trac* and *Cd8a*, the IgM receptor gene *Fcmr*, and the tumor necrosis factor family member lymphotoxin-β gene (*Ltb*) that functions in GC formation (46), all of which are DEGs in SjD vs. HV MSGs (Figure 4B and Supplementary Table S1). Also identified was the upregulation of the lncRNA gene *Gm15987* and *Fam169b* and the downregulation of the transcription factor *Dbp*, which were not identified as DEGs in SjD vs. HV MSGs. Gene ontology analysis of upregulated unique IL-14αTG mouse SMG DEGs identified 215 significantly enriched biological processes and 55 KEGG pathways in total, with 81 biological processes and 44 KEGG pathways shared in IL-14αTG mouse and human SjD samples (Figure 4C and Supplementary Table S1). In contrast to human and mouse diseased vs. control transcriptome comparisons, the unique IL-14αTG mouse SMG downregulated DEGs showed significant enrichment of the circadian rhythm and rhythmic process KEGG pathway and biological processes (Figure 4D).

### Spatial transcriptomic analysis revealed tissue localization of immune cell gene markers

**Figure 5:**
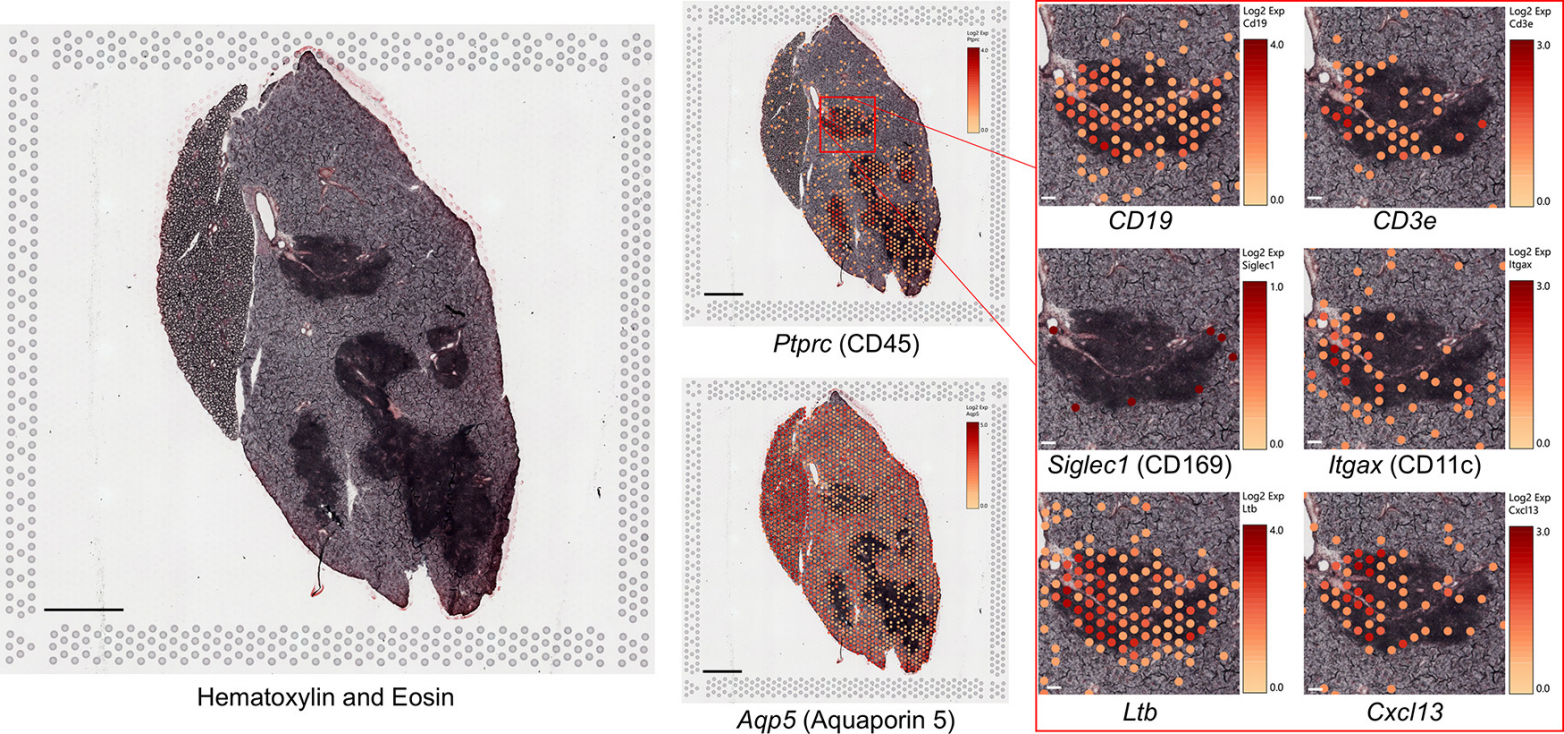
Spatially resolved gene marker expression in IL-14αTG mouse salivary glands using spatial transcriptomic analysis. Fresh frozen SMG and SLG cryosections from a 12-month-old female IL-14αTG mouse were adhered to a barcoded Visium spatial gene expression slide, stained with hematoxylin and eosin, visualized on a Zeiss Axiovert 200M inverted microscope, and then subjected to RNAseq analysis. Color-coded expression of individual gene markers [Log2(counts/capture area)] in barcoded mRNA capture areas was overlaid on the tissue image using Loupe browser 8.1 software. Scale bar = 1 mm and 100 µm (inset).

Results from spatial transcriptomic analysis validated immune cell marker localization observed by immunofluorescence analysis and provided insights on tissue localization of DEGs identified by RNAseq analysis. In 12-month-old IL-14αTG mouse SMG and SLG, localization of mRNA encoding the salivary acinar cell water channel aquaporin 5 (*Aqp5*) and the pan-immune cell marker CD45 (*Ptprc*) (Figure 5) largely mirrored observations from immunofluorescence analysis of a serial IL-14αTG mouse salivary gland section using anti-AQP5 and anti-CD45 antibodies (Figure 1C). *Aqp5* and *Ptprc* expression delineated salivary acinar tissue and immune cell foci, respectively. However, *Aqp5* expression was also observed within the immune cell foci and immune cells did not ubiquitously express *Ptprc* in contrast to immunofluorescence observations. The *Aqp5* localization in immune cells may reflect the presence of acinar cells localized above or below the focal plane during confocal microscopy and the lack of *Ptprc* expression in immune cells may be due to low-level mRNA expression that was below the sequencing threshold. Visium v1 uses a grid of barcoded probe capture areas 55 µm in diameter that bind polyadenylated mRNA, resulting in each cluster being a representative mRNA pool from multiple cell types in the capture area. When examining localization of immune cell marker genes, many immune cell clusters expressed gene markers for multiple cell types. Because the B cell marker antibody B220 (CD45R) detects a splice variant of *Ptprc*, we instead used *CD19* expression to identify B cells and *CD3e* expression to identify T cells and observed distinct expression patterns within each focus (Figure 5). Localization of *Siglec1* (CD169) and *Itgax* (CD11c) resembled immunofluorescence analysis, where CD169^+^ macrophages localized to the focus periphery and CD11c^+^ dendritic cells were present throughout the focus (Figure 1C). The GL7 antibody used for the detection of activated B and T cells in germinal centers binds a sialic acid glycan (47), so we instead observed the localization of *Ltb* and *Cxcl13* that are essential, but not exclusive, markers for GC development (46, 48) and found robust expression of both genes in IL-14αTG mouse salivary gland immune cell foci (Figure 5).

### Clustering of spatial transcriptomic capture areas revealed cell type enrichment and localization within salivary gland tissue

**Figure 6:**
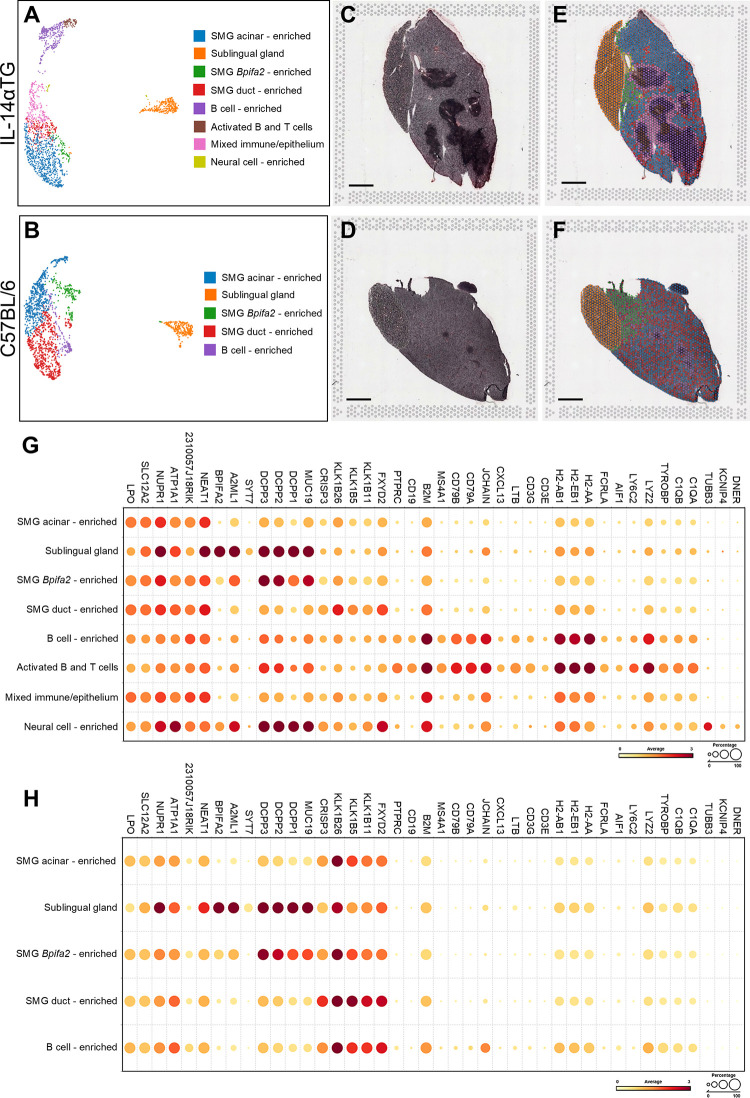
Spatial transcriptomic analysis and cell clustering of IL-14αTG and C57BL/6 mouse salivary glands. RNAseq analyses of barcoded Visium spatial mRNA capture areas from 12-month-old female IL-14αTG or C57BL/6 mouse SMG and SLG were subjected to unsupervised Louvain clustering and visualization using Bioturing software. **(A,B)** UMAP and color-coded cell cluster identification, **(C,D)** hematoxylin and eosin (H&E)-stained tissue images, **(E,F)** cell cluster spatial localization overlay on H&E tissue images, and **(G,H)** dot plot of cell cluster-defining gene markers in **(A,C,E)** IL-14αTG and **(B,D,F)** C57BL/6 mouse SMG and SLG. Scale bar = 1 mm.

We utilized BioTuring software to perform clustering of mRNA capture areas and identify areas spatially enriched in specific cell types (Figure 6). Unsupervised clustering of capture areas identified 9 distinct cell clusters for the 12-month-old IL-14αTG mouse SMG and SLG and 7 clusters for the C57BL/6 mouse salivary glands (data not shown). However, two IL-14αTG and three C57BL/6 mouse clusters were enriched in salivary gland acinar genes and appeared otherwise indistinguishable. Therefore, these acinar-enriched clusters were combined for each sample and the uniform manifold approximation and projection (UMAP) dimensionality reduction revealed a total of 8 IL-14αTG (Figure 6A) and 5 C57BL/6 (Figure 6B) mouse salivary gland cell clusters. Color-coded, cell type-enriched clusters were overlayed on the hematoxylin and eosin-stained salivary gland sections (Figures 6C,D) to reveal spatially resolved cell type enrichment (Figures 6E,F). Due to the low resolution of Visium v1 resulting from 55 µm diameter probe capture areas, identification of individual cell types was not possible, and cluster annotations indicate enrichment signatures for the indicated cell types rather than homogeneous cell populations. For cluster annotation, we first utilized the cluster-defining genes from a previously published mouse SMG scRNAseq analysis (25). However, many of the cell type gene markers used for annotation, such as acinar cell secretoglobins (*i.e.*, *Scgb2b26, Scgb1b27* and *Scgb2b27),* were ubiquitously expressed across all clusters (Supplementary Figure S5). Furthermore, the scRNAseq dataset lacked representative sublingual gland, B cell, T cell and neural cell populations. Instead, clusters were annotated using published mouse salivary and lacrimal gland scRNAseq and spatial transcriptomic datasets (25, 26, 49) and the cell type prediction tool of BioTuring SpatialX that uses a reference database of all annotated scRNAseq datasets in the Talk2Data module. In both IL-14αTG and C57BL/6 mouse tissues, sublingual glands formed a single cluster marked by *Dcpp1-3*, *A2ml1* and *Syt7*, whereas submandibular glands contained multiple cell clusters enriched in salivary epithelial cell markers and/or immune cell markers (Figures 6G,H). SMG acinar cell markers included *Lpo*, *Slc12a2* and *Nupr1* while SMG duct cell markers included *Crisp3* and various kallikrein serine proteases (*i.e.*, *Klk1b26*, *Klk1b5* and *Klk1b11*). In the SMG of both genotypes, a population of histologically epithelial cell clusters located near the sublingual gland were enriched in *Bpifa2*, *Dcpp1*-3 and *Muc19* compared to SMG acinar- and SMG duct-enriched clusters and these likely represent *Bpifa2*^+^ serous-like acinar cells previously identified in the adult mouse SMG (25). In the C57BL/6 mouse SMG, one cell cluster was enriched in immune cells including the B cell markers *Jchain, CD79A* and *CD79B* and the macrophage markers *C1Qa/b*, *Tyrobp* and *H2-Aa*, with lower expression of the T cell marker *CD3e*. In the IL-14αTG mouse SMG, 3 cell clusters were enriched in immune cell gene markers including the B cell-enriched cluster found in C57BL/6 mouse SMG, an activated B and T cell cluster with increased expression of *CD3e* and the GC markers *Cxcl13* and *Ltb*, and a mixed immune/epithelial cell cluster located at the periphery of the immune cell foci. The latter cell cluster appears to be SMG acinar and ductal cells histologically, but also expresses higher levels of immune cell markers compared to SMG acinar-enriched and duct-enriched cell clusters. Lastly, cell clusters located near the main excretory duct in the IL-14αTG mouse SMG expressed the neuronal microtubule component gene *Tubb3*, the voltage-gated potassium channel-interacting protein gene *Kcnip4*, and the dendritic notch signaling regulator gene *Dner*, thusly defining a population of neural cells that likely represent the SMG-innervating facial nerve that is closely associated with the Wharton's excretory duct and blood vessels that vascularize the SMG (50).

### Spatial localization and cell cluster enrichment of gene markers for immune cell subtypes and DEGs

**Figure 7:**
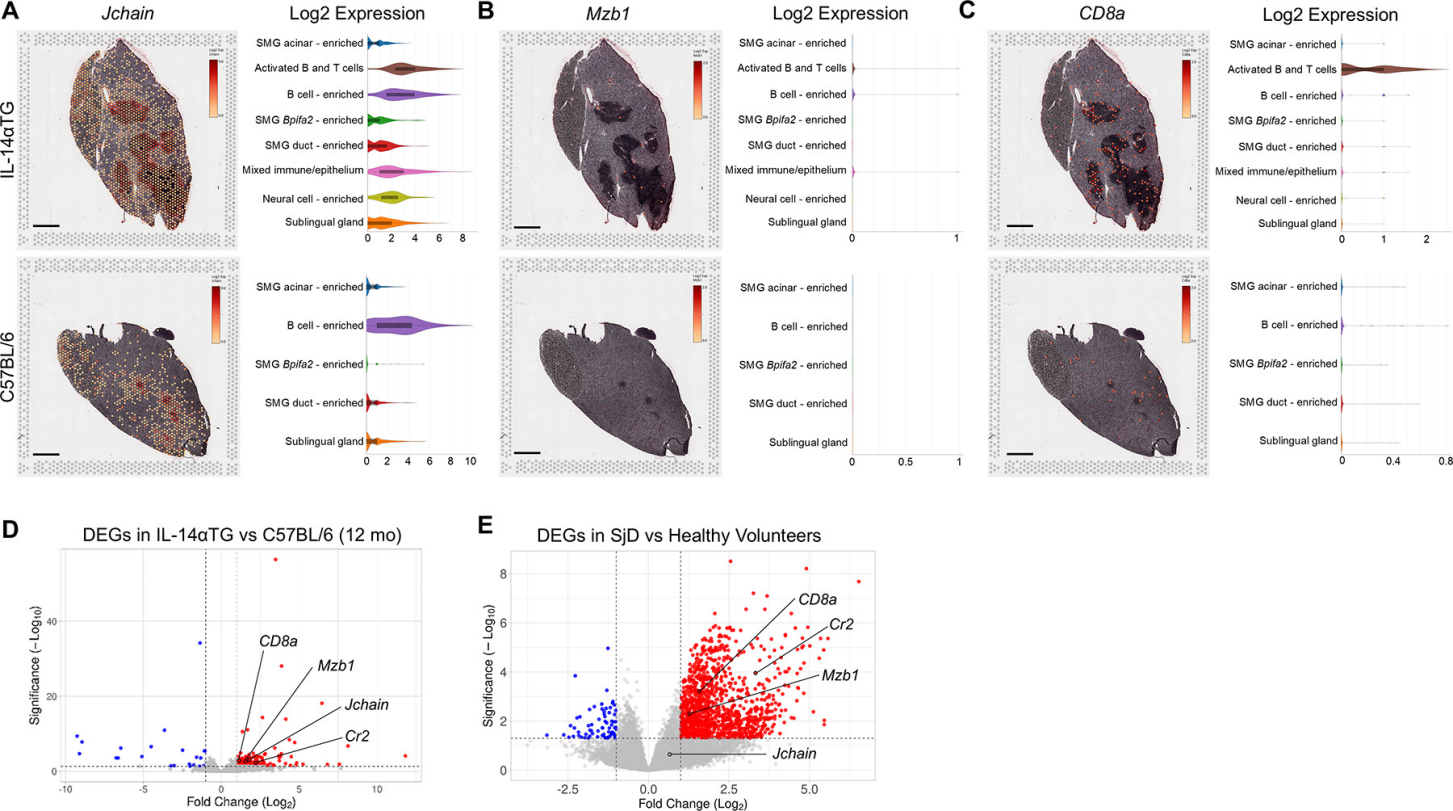
Immune cell gene marker expression, localization, and cell cluster distribution in IL-14αTG and C57BL/6 mouse salivary glands. Color-coded expression [Log2(counts/capture area)] and cell cluster distribution violin plots of **(A)** *Jchain* (plasma cell marker), **(B)** *Mzb1* (MZ B cell marker) and **(C)** *CD8a* (CD8 T cell marker) in 12-month-old IL-14αTG and C57BL/6 mouse SMG and SLG; scale bar = 1 mm. Volcano plots denoting immune cell marker DEGs identified by RNAseq analysis of **(D)** 12-month-old IL-14αTG vs. C57BL/6 mouse SMG and **(E)** SjD patient vs. healthy volunteer MSG.

Flow cytometry results indicated that plasma cells represented the largest population of CD45^+^ immune cells in both IL-14αTG and C57BL/6 mouse SMG (Figure 2D) and these cells abundantly express *Jchain*, a linker polypeptide for the multimeric assembly of IgM and IgA from antibody-secreting cells (51). Spatial transcriptomic analysis showed high *Jchain* expression localized in and around the SMG immune cell foci of both genotypes and violin plots demonstrated enriched expression in activated B and T cell, B cell-enriched, and mixed immune/epithelium cell clusters, with lower expression in non-immune cell clusters (Figure 7A). In the IL-14αTG mouse salivary gland, higher level *Jchain* expression was observed at the periphery of immune cell foci, with the core showing lower or no expression. The proportion of marginal zone B cells was significantly increased in IL-14αTG, as compared to C57BL/6 mouse SMG (Figure 2C), and MZ B cells abundantly express *Mzb1*, which encodes the endoplasmic reticulum protein pERp1 that functions in Ca^2+^ homeostasis and immunoglobulin assembly (52). In the SMG, low levels of *Mzb1* were expressed in IL-14αTG mice with spatial localization in immune cell-enriched clusters, but *Mzb1* was undetectable in C57BL/6 mice (Figure 7B). Similarly, mRNA capture areas co-expressing the MZ B cell markers *CD19* and *Cr2* (*i.e.*, complement receptor 2; CD21/35) were identified in IL-14αTG mouse SMG, but were absent in C57BL/6 mouse SMG (Supplementary Figure S6). While the proportion of CD8^+^ T cells was significantly increased in C57BL/6 mouse SMG, as compared to IL-14αTG mice (Figure 2F), absolute expression of the CD8 alpha subunit gene *CD8a* was greater in IL-14αTG mouse SMG and violin plots show localization primarily in the activated B and T cell cluster with low expression in all other cell clusters (Figure 7C). All four immune cell marker genes *Jchain*, *Mzb1*, *Cr2*, and *CD8a* were upregulated DEGs in RNAseq analyses of 12-month-old IL-14αTG vs. C57BL/6 mouse SMG (Figure 7D) and *Mzb1*, *Cr2* and *CD8a* were upregulated DEGs in human SjD vs. healthy volunteer MSG (Figure 7E).

**Figure 8:**
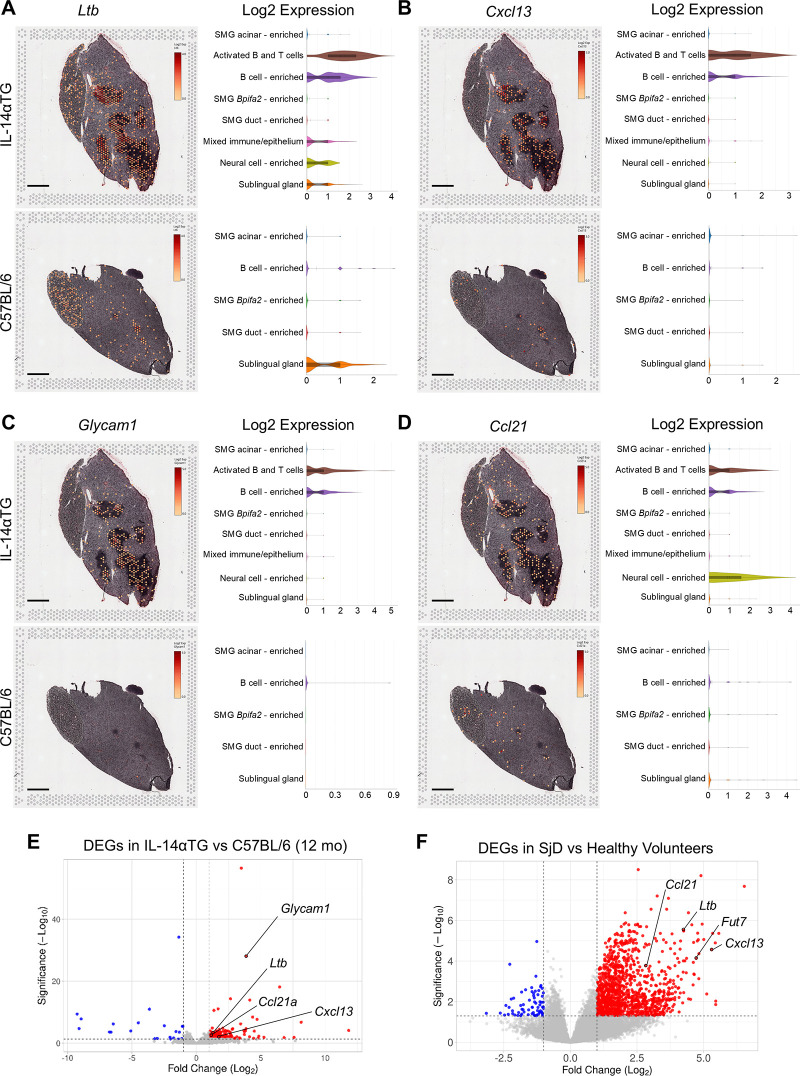
Expression, spatial localization and cell cluster distribution of germinal center and high endothelial venule gene markers in IL-14αTG and C57BL/6 mouse salivary glands. Color-coded expression [Log2(counts/capture area)] and cell cluster distribution violin plots of **(A)** *Ltb* (lymphotoxin-β, GC gene marker), **(B)** *Cxcl13* (C-X-C motif chemokine ligand 13, GC gene marker), **(C)** *Glycam1* (glycosylation dependent cell adhesion molecule 1, HEV gene marker) and **(D)** *Ccl21* (C-C motif chemokine ligand 21, HEV gene marker) in 12-month-old IL-14αTG and C57BL/6 mouse SMG and SLG; scale bar = 1 mm. Volcano plots denoting GC and HEV DEG markers identified by RNAseq analysis of **(D)** 12-month-old IL-14αTG vs. C57BL/6 mouse SMG and **(E)** SjD patient vs. healthy volunteer MSG.

Flow cytometry analysis identified GL7^+^ GC B cells in both 12-month-old IL-14αTG and C57BL/6 mouse SMG (Figure 2C) and we observed expression of the GC gene markers *Ltb* and *Cxcl13* in immune cell foci in IL-14αTG mouse SMG (Figure 5). Spatial transcriptomic analysis revealed abundant expression of *Ltb* in IL-14αTG compared to C57BL/6 mouse SMG that was primarily localized to activated B and T cell and B cell-enriched clusters, with lower expression observed in mixed immune/epithelium, neural cell-enriched and sublingual gland clusters (Figure 8A). Similarly, *Cxcl13* was more abundantly expressed in IL-14αTG compared to C57BL/6 mouse SMG and expression was almost exclusively localized to activated B and T cell and B cell-enriched clusters (Figure 8B). RNAseq analysis of 12-month-old IL-14αTG vs. C57BL/6 mouse SMG indicated that *Glycam1,* a marker for high endothelial venules (HEVs), was one of the most highly upregulated DEGs based on Manhattan distance from origin (Figure 3C). Whereas GCs serve as sites for B cell maturation and clonal expansion, HEVs support the extravasation of lymphocytes from the blood stream into inflamed tissues (53). *Glycam1* was abundantly expressed in the activated B and T cell and B cell-enriched clusters of IL-14αTG mouse SMG, but was nearly undetectable in C57BL/6 mouse SMG (Figure 8C). Similarly, *Ccl21a* encoding the potent lymphocyte chemokine expressed in HEV endothelial cells (54), was highly expressed in activated B and T cell and B cell-enriched clusters of IL-14αTG mouse SMG, with expression also observed in the neural cell-enriched cluster (Figure 8D). RNAseq analysis further identified *Ltb*, *Cxcl13* and *Ccl21a* as upregulated DEGs in IL-14αTG vs. C57BL/6 mouse SMG (Figure 8E) and in human SjD vs. HV MSG (Figure 8F). While *Glycam1* is a non-expressed pseudogene in humans, another HEV marker involved in lymphocyte migration, *Fut7* (fucosyltransferase 7) (54), was also significantly upregulated in SjD MSG (Figure 8F).

**Figure 9:**
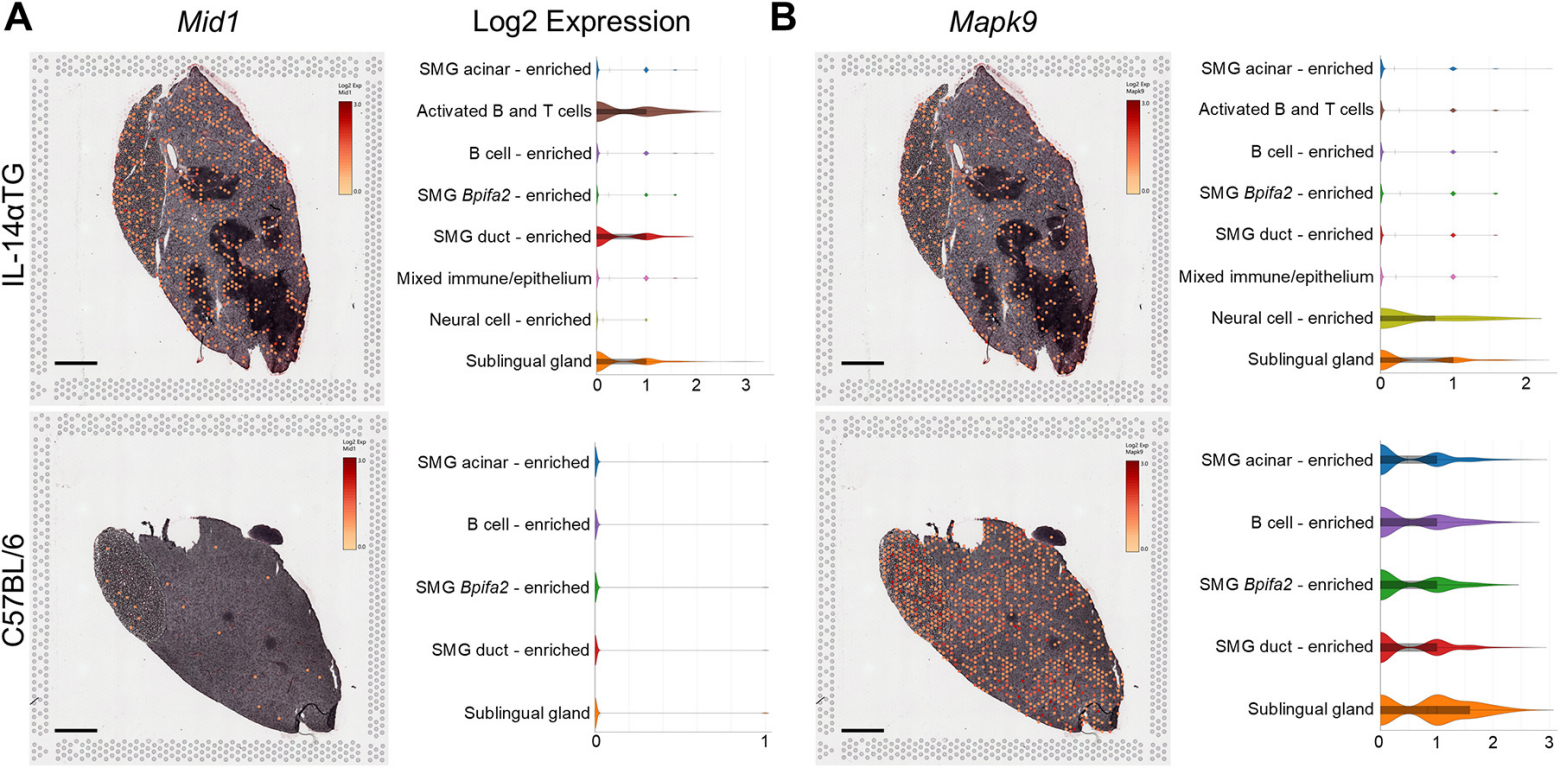
*Mid1* and *Mapk9* expression, spatial localization and cell cluster distribution in IL-14αTG and C57BL/6 mouse salivary glands. Color-coded expression [Log2(counts/capture area)] and cell cluster distribution violin plots of **(A)** *Mid1* (midline 1; TRIM18) and **(B)** *Mapk9* (mitogen-activated protein kinase 9; c-Jun N-terminal kinase 2, JNK2) in 12-month-old IL-14αTG and C57BL/6 mouse SMG and SLG; scale bar = 1 mm.

The most significantly upregulated and downregulated DEGs identified by RNAseq of 12-month-old IL-14αTG mouse SMG were the microtubule-associated protein gene *Mid1* and the mitogen-activated protein kinase gene *Mapk9*, respectively (Figure 9). Both *Mid1* and *Mapk9* were also identified as DEGs in IL-14αTG mouse SMG at the 6-month timepoint (Supplementary Table S1) and *Mid1* was identified as a unique SMG DEG that was differentially expressed with age in IL-14αTG, but not in C57BL/6 mouse SMG. Spatial transcriptomic analysis supported RNAseq findings, with broad *Mid1* expression observed in all cell clusters and enriched in activated B and T cell, SMG duct cell-enriched and sublingual gland cell clusters in the IL-14αTG mouse, whereas *Mid1* expression in C57BL/6 mouse SMG and SLG was sparse in all cell clusters (Figure 9A). *Mapk9* expression was abundant in C57BL/6 mouse SMG and SLG and localized to all cell clusters, whereas *Mapk9* localization in IL-14αTG salivary glands was noted in all clusters, but was most abundant in neural cell-enriched and sublingual gland cell clusters (Figure 9B). Neither *Mid1* nor *Mapk9* were identified as a DEG in human SjD MSG (Supplementary Table S1).

## Discussion

One key aspect of chronic human diseases is the lag time between disease onset and clinical diagnosis. While Sjögren's disease is typically diagnosed in the 4th or 5th decade of life, many patients experience disease symptoms for years prior to diagnosis. As a result, clinical research samples are skewed towards advanced stages of disease, making it difficult to parse molecular mechanisms that contribute to early disease initiation. Therefore, identifying early drivers of disease is critical for enabling earlier diagnosis and intervention. Because the IL-14αTG mouse model has a well-defined temporal progression of SjD-like disease pathology, we can utilize both pre-symptomatic and post-symptomatic animals to compare molecular mechanisms of SjD and how they may be targeted therapeutically. Utilizing transcriptomic analyses to identify signaling pathways that are up or down regulated during disease progression could identify novel druggable targets or early diagnostic biomarkers that can be experimentally investigated in IL-14αTG mice at early or late stages of disease and then validated using human samples.

We investigated transcriptomic and histological changes in IL-14αTG and age-matched control C57BL/6 mouse SMG at 6 and 12 months of age, corresponding to time points preceding and subsequent to the development of sialadenitis in IL-14αTG mice (16, 18). The selection of comparative time points is of great importance in SjD-like mouse models due to the wide temporal range in disease development. In some SjD mouse models such as the NOD.B10-H2^b^ and C57BL/6.NOD-*Aec1Aec2* mice, salivary gland dysfunction and sialadenitis can be detected as early as 12 weeks of age (55, 56), whereas SjD-like pathologies appear at 12–14 months of age in BAFF/BLyS transgenic mice (57). In IL-14αTG mice, hypergammaglobulinemia can be detected in blood serum as early as 12 weeks of age, but salivary gland dysfunction (*i.e.*, loss of saliva secretion) is not measurable until 6 months of age and precedes the development of salivary and lacrimal gland inflammation that occurs at 9–12 months of age (16, 18, 58). IL-14αTG mice were generated on a C57BL/6 background and previous studies have demonstrated that aged C57BL/6 mice also develop salivary and lacrimal gland inflammation by 12–24 months of age (38, 45). Indeed, our RNAseq comparison of 12-month-old vs. 6-month-old C57BL/6 mouse SMG showed significant enrichment of inflammatory pathways and biological processes that occur during aging (Supplementary Table S1). However, even after eliminating age as a variable by comparing age-matched 12-month-old IL-14αTG vs. C57BL/6 mouse SMG, we still observed significant enrichment of DEGs involved in inflammatory pathways and biological processes and our immunofluorescence and flow cytometry analyses further confirm the enhanced salivary gland inflammation in IL-14αTG mice compared to control (Figures 1–3). We further controlled for age-associated inflammatory changes in C57BL/6 mice in our SMG RNAseq data by identifying and removing genes that were differentially expressed with age in both IL-14αTG and C57BL/6 genotypes to generate a list of DEGs that were unique to the IL-14αTG mouse SMG (Figure 4). Again, gene ontology analysis identified significant enrichment of inflammatory and immune pathways that heavily overlapped with gene changes that occur in MSG biopsies of human SjD patients vs. healthy volunteers. Age-associated differential gene expression in salivary gland also likely occurs in human SjD patients and, considering that the disease is typically diagnosed between 40 and 55 years of age (4), age-associated gene expression should be considered as a confounding variable in future SjD studies and clinical trials. In the SjD vs. healthy volunteer MSG RNAseq dataset accessed through dbGAP (accession phs001842.v1.p1) and analyzed here, the median age of SjD patients at the time of biopsy was 52 while the median age of healthy volunteers was 29 (20).

The role of B cells in SjD pathogenesis has long been a focus of clinical investigation and B cell targeting therapies remain one of the few therapeutic options for severe or refractory SjD patients after failure of traditional treatment regimens (5, 59). Previous studies have demonstrated significantly increased levels of germinal center, plasma and marginal zone B cells in the spleen of IL-14αTG mice compared to littermate controls, as well as increased B1 cell levels in the peritoneal cavity (16, 58). In contrast to conventional B2 cell subsets that carry out humoral immunity, B1 cells are innate-like B cells primarily found in the peritoneal and pleural cavities that predominate during neonatal development (60). B1 cells possess the ability for self-renewal, generate the majority of IgM and IgA and have been shown to produce anti-phosphatidylserine and anti-dsDNA antibodies in systemic lupus erythematosus (SLE) and autoimmune diabetes mouse models (61, 62). Our data with SMG-infiltrating immune cells demonstrate that B1 cells are a minor B cell population in both IL-14αTG and C57BL/6 mouse SMG (∼5%) and no statistical difference was observed between genotypes with regards to total B1 cells, CD5^+^ B1a cells that produce broadly reactive IgM, or CD5^−^ B1b cells that produce a T cell-independent, long lasting memory type of IgM (41, 60). Marginal zone B cells possess innate-like immune cell qualities similar to B1 cells, but also participate in conventional humoral immune responses (63). While typically located in the splenic marginal zone between the white pulp and blood circulation, MZ B cells have been identified in MSG biopsies of SjD patients and in lymph nodes of SLE patients (64, 65). Unlike B1 cells, MZ B cells have been shown to play a central role in the development of the IL-14αTG mouse phenotype, where ablation of MZ B cells in IL-14αTG mice prevented the development of sialadenitis and salivary dysfunction, whereas ablation of B1 cells had no effect on disease phenotype (58). Our data demonstrate an increased proportion of MZ B cells in IL-14αTG compared to C57BL/6 mouse SMG, in line with previous analyses of splenic B cells (16). We further demonstrate increased expression of the MZ B cell marker gene *Mzb1* in both IL-14αTG mouse SMG and human SjD MSG (Figure 7). MZ B cells were also identified by co-expression of *CD19* and CD21/35 (*Cr1* and *Cr2*; complement receptor 1 and 2) using spatial transcriptomics (Supplementary Figure 6) and RNAseq analysis identified *Cr1* and *Cr2* as upregulated DEGs in both IL-14αTG mouse SMG and human SjD MSG (Supplementary Table S1). Interestingly, these complement receptors serve as the cell surface receptor for Epstein–Barr virus (EBV) that exhibits tropism for B cells and has been suggested as an initiating factor in SjD pathogenesis (2, 66). While specific contributions of MZ B cells to salivary gland dysfunction have not been clinically demonstrated in SjD patients, most SjD-associated lymphomas are MZ B cell neoplasms that include the MALT lymphoma subset (67, 68).

For other conventional B2 cell subsets, flow cytometry data showed no difference in proportional levels of SMG-infiltrating germinal center, plasma, follicular or memory B cells between genotypes at 12 months of age (Figure 2C-D). However, total CD45^+^ immune cells were significantly increased in IL-14αTG mouse SMG (Figure 2A) and RNAseq analysis identified numerous immune cell gene markers that were significantly upregulated compared to C57BL/6 mouse SMG, despite there being no difference in the proportion to total immune cells between genotypes. Among all immune cell types analyzed, plasma cells were the most abundant in both genotypes and the plasma cell gene marker *Jchain* was a significantly upregulated DEG in IL-14αTG vs. C57BL/6 mouse SMG (Figure 7). These terminally differentiated antibody secreting B cells arising from either B1 or conventional B2 cell compartments mediate humoral immune responses under homeostatic conditions; however, plasma cells may also contribute to chronic autoinflammatory responses in SLE and SjD through autoantibody production (69). Plasma cells are typically resistant to B cell depletion therapies as they are non-dividing cells that lack CD20 expression, which may explain the low efficacy of B cell depletion therapies in SjD patients (69). When sorted by fold-change (log2FC), immunoglobulin genes represent 19 of the top 20 upregulated DEGs in the 12-month-old IL-14αTG mouse SMG (Supplementary Table S1), further highlighting the significant increase in antibody production that occurs in this SjD mouse model. Although flow cytometry indicated a lower proportion of CD8^+^ T cells in IL-14αTG vs. C57BL/6 mouse SMG (Figure 2F), RNAseq analysis identified the CD8 T cell gene marker *CD8a* as a significantly upregulated DEG in IL-14αTG mouse salivary gland and spatial transcriptomic analysis showed abundant *CD8a* expression in the activated B and T cell cluster (Figure 7).

Similarly, the proportion of SMG-infiltrating GL7^+^ GC B cells was not significantly different between genotypes (Figure 2C); however, the GC markers *Ltb* and *Cxcl13* were upregulated DEGs in 12-month-old IL-14αTG vs. C57BL/6 mouse SMG and highly expressed in the activated B and T cell and B cell-enriched clusters of IL-14αTG SMG (Figure 8). Germinal centers are typically found in secondary lymphoid tissue such as spleen and lymph nodes where they develop in response to antigen recognition by B cells that migrate through a specialized network of follicular dendritic cells and T cells to undergo affinity-dependent selection and clonal expansion (39). GC formation in non-lymphoid tissues also occurs during autoimmune disease pathogenesis and spontaneous ectopic GC formation is a hallmark of many autoimmune disease mouse models (70). Spontaneous GC-like lymphoid structures and GL7^+^ GC B cells have also been described in 13-14-month-old C57BL/6 mouse SMG (45) and our data further support the concept of spontaneous sialadenitis, GC formation and subsequent loss of salivary gland function in aged C57BL/6 mouse exocrine tissues. In the IL-14αTG mouse model, these age-associated immune responses are compounded such that even after controlling for age as a variable, we still observe significantly increased levels of GC gene markers *Ltb* and *Cxcl13*. In human SjD MSG, gene ontology analysis of DEGs identified GC formation as an enriched biological process, with the upregulation of GC-associated genes Tnfsf13b (BAFF/BlyS), *Ada*, *Klhl6*, and *Unc13d* (Supplementary Table S1). While previous studies suggest that only ∼25% of SjD patient MSG biopsies contain GC-like structures at the time of diagnosis, their presence may have prognostic value and is associated with an ∼8-fold increase in the risk of developing non-Hodgkin lymphoma (8, 71).

While our flow cytometric and transcriptomic analyses focused on lymphocytes and adaptive immune responses, innate immune processes and interferon (IFN) signaling also contribute to salivary gland dysfunction in SjD (72). Elevated IFN-inducible gene expression has been observed in SjD patient salivary glands and peripheral blood mononuclear cells (73, 74) and single nucleotide polymorphisms associated with SjD development have been identified in numerous IFN-inducible genes involved in innate immunity, including interferon regulatory factor 5 (*Irf5*), signal transducer and activator of transcription 4 (*Stat4*) and human leukocyte antigen (HLA) genes (75). Bulk RNAseq analysis identified numerous differentially expressed IFN-inducible genes, including *Irf5*, *Irf8*, *Ifit3b* and *Ifi47* in 12-month-old IL-14αTG vs. C57BL/6 SMGs and *Irf8*, *Irf1*, *Ifit3* and *Ifi44* in SjD vs. HV MSGs (Supplementary Table S1). Furthermore, the toll-like receptor (TLR) genes *Tlr7*, *Tlr9* and *Tlr10* were also identified as DEGs in both human and mouse datasets, suggesting that innate immune responses contribute to SjD pathogenesis (Supplementary Table S1).

In addition to immune cell and GC gene markers, RNAseq analysis identified the high endothelial venule gene marker *Glycam1* as one of the top DEGs in 12-month-old IL-14αTG compared to C57BL/6 mouse SMG and spatial transcriptomic analysis revealed abundant expression of *Glycam1* and the HEV-associated chemokine *Ccl21* in the activated B and T cell and B cell-enriched clusters of IL-14αTG mouse SMG (Figure 8). HEVs are specialized blood vessel structures that promote lymphocyte trafficking from the bloodstream into surrounding tissue through expression of various cell adhesion molecules, selectins, chemokines and enzymes (53). Like GCs, HEVs are typically found in secondary lymphoid tissues where they function in homeostatic immunosurveillance, but have also been identified in chronically inflamed non-lymphoid tissues in autoimmune diseases, including synovium in rheumatoid arthritis and salivary glands in SjD patients (76). *Glycam1*^+^ HEVs have been identified in inflamed salivary and lacrimal glands of NOD mice, where lymphotoxin-β receptor signaling was essential for their development (77, 78), as well as in 24-month-old C57BL/6 mouse lacrimal glands (38), further highlighting the overlap of age-associated inflammatory processes and autoimmune-mediated pathologies. While *Glycam1* is a pseudogene in humans, our RNAseq analysis of SjD patient MSGs identified two other HEV gene markers, *Fut7* and *Ccl21* (54), as upregulated DEGs compared to healthy volunteer MSGs (Figure 8). Because HEV formation is preceded by tissue lymphocyte accumulation and is often associated with ectopic GCs (76), HEVs aren't likely to be a causative agent for chronic inflammation, but may represent a therapeutic target to reduce lymphocyte trafficking into inflamed tissue.

RNAseq analysis identified *Mid1* and *Mapk9* as the top upregulated and downregulated DEGs, respectively, in 12-month-old IL-14αTG vs. C57BL/6 mouse SMG (Figure 3C) and *Mid1* and *Mapk9* were 2 of 9 total genes that were differentially expressed in IL-14αTG mouse SMG at both the 6- and 12-month time points (Supplementary Table S1). *Mid1* encodes a member of the interferon-inducible tripartite motif family of proteins, (TRIM18; midline 1), and mutations in this cytoplasmic microtubule-associated protein are causally implicated in X-linked Opitz G/BBB syndrome characterized by abnormalities such as cleft lip, heart defects and agenesis of the corpus callosum (79). Mid1 exhibits E3 ubiquitin ligase activity targeting the Alpha4 (α4) protein (80), a microtubule-associated protein regulatory subunit originally identified as an Ig receptor binding protein that contributes to B cell receptor signaling (81). By regulating ubiquitin-mediated degradation of α4, Mid1 modulates the assembly of the mammalian target of rapamycin complex 1 (mTORC1) that has been investigated for treatment of lacrimal and salivary gland pathologies in SjD patients (82–84). *Mapk9* encodes c-Jun N-terminal kinase 2 (JNK2), a primary regulator of the c-Jun component of the activator protein-1 (AP-1) family of transcription factors, which has been shown to contribute to peripheral T cell activation in response to extracellular cytokines and proinflammatory activators (85, 86). JNK2 knockout confers protection against insulitis and diabetes development in NOD mice and pharmacological JNK2 inhibition increased tear production in the MRL/lpr mouse model of SjD (87, 88). In the NOD.B10-H2^b^ SjD mouse model, bulk RNAseq analysis of SMGs identified *Mapk9* as an upregulated DEG compared to C57BL/10 control mice at 7 months of age and scRNAseq demonstrated *Mapk9* enrichment in the excretory duct and NK cell compartments (27). The discrepancy between downregulation and upregulation of *Mapk9* expression in IL-14αTG and NOD.B10-H2^b^ mice, respectively, likely reflects the heterogeneity of SjD mouse models and the relative contributions of B and T lymphocytes to SMG pathologies. Neither *Mid1* nor *Mapk9* were identified as DEGs in SjD MSG biopsies; however, alternative gene products that carry out similar cellular functions were identified, including the gene encoding the microtubule-associated protein TRIM46 that functions in neuronal axon specification and polarity (89) and *Mapk12* encoding p38γ that negatively regulates c-Jun expression and subsequent AP-1 transcription factor activity (90).

In summary, we assessed chronic autoimmune sialadenitis in IL-14αTG mouse SMG using immunofluorescence, flow cytometry, bulk RNAseq and spatial transcriptomic analyses and compared the results to previous findings in salivary glands of SjD mouse models and minor salivary gland biopsies from human SjD patients. We highlighted the overlapping inflammatory signatures of DEGs in SMGs of age-matched IL-14αTG vs. control C57BL/6 mice and MSGs of SjD patients vs. healthy volunteers, while also confirming previous observations of age-associated inflammatory changes in mouse salivary and lacrimal glands. One of the limitations of this study is the disease stage of the tissue samples that were analyzed. In mouse tissues, the C57BL/6 control group exhibited significant age-associated inflammatory changes that were difficult to parse from autoimmune-mediated inflammatory changes. Further examination of the SjD-like phenotype of IL-14αTG mice from 3 to 9 months of age, corresponding to asymptomatic and mild symptomatic time points when tissues are less inflamed, may offer further mechanistic insight into disease-initiating factors that can be targeted therapeutically. In the human RNAseq dataset, the median age difference of SjD patients and healthy volunteers at the time of MSG biopsy represents an uncontrolled variable that should be considered when scrutinizing our findings in humans. Limitations of the arrayed poly(dT)-based spatial transcriptomic analysis include low resolution resulting from the 55 µm diameter capture areas and the high dependence on the tissue sections chosen for comparison. The large capture areas encompassing multiple cell types and the resulting pool of barcoded mRNA from these cells make cell clustering challenging. The SMG and SLG tissue sections chosen for comparison can further impact experimental effectiveness and reproducibility due to the high spatial heterogeneity of salivary glands. For example, the 10 µm IL-14αTG mouse salivary tissue section analyzed here by spatial transcriptomics included a large portion of the main excretory duct and facial nerve, whereas the C57BL/6 tissue section did not contain either. Similarly, clusters of activated B and T cells were not identified in the C57BL/6 mouse SMG and it is unclear whether these cells were not present in the intact salivary gland or just absent in the chosen tissue section. Nonetheless, the complementary use of whole tissue RNAseq analysis offered clarity in identifying upregulated gene markers of activated B and T cells (*i.e.*, *Ltb* and *Cxcl13*) in the IL-14αTG mouse SMG, giving greater confidence that these cell types are more abundant in this SjD-like mouse compared to control C57BL/6 mouse SMG. Despite technical limitations, the use of unbiased bulk and spatial transcriptomic profiling to refine interpretations of existing data offers a powerful approach to support hypothesis-driven research.

## Data Availability

The datasets presented in this study can be found in online repositories. The names of the repository/repositories and accession number(s) can be found in the article/Supplementary Material.
