# Supplementary material for: Salivary gland transcriptomic analysis and immunophenotyping in the IL-14α transgenic mouse model of Sjögren's disease
Source: Front Dent Med. 2025 Jul 8;6:1612522. doi: 10.3389/fdmed.2025.1612522 (PMC12279800; doi:10.3389/fdmed.2025.1612522)
Supplement: Supplementary file 1 [file Datasheet1.docx]

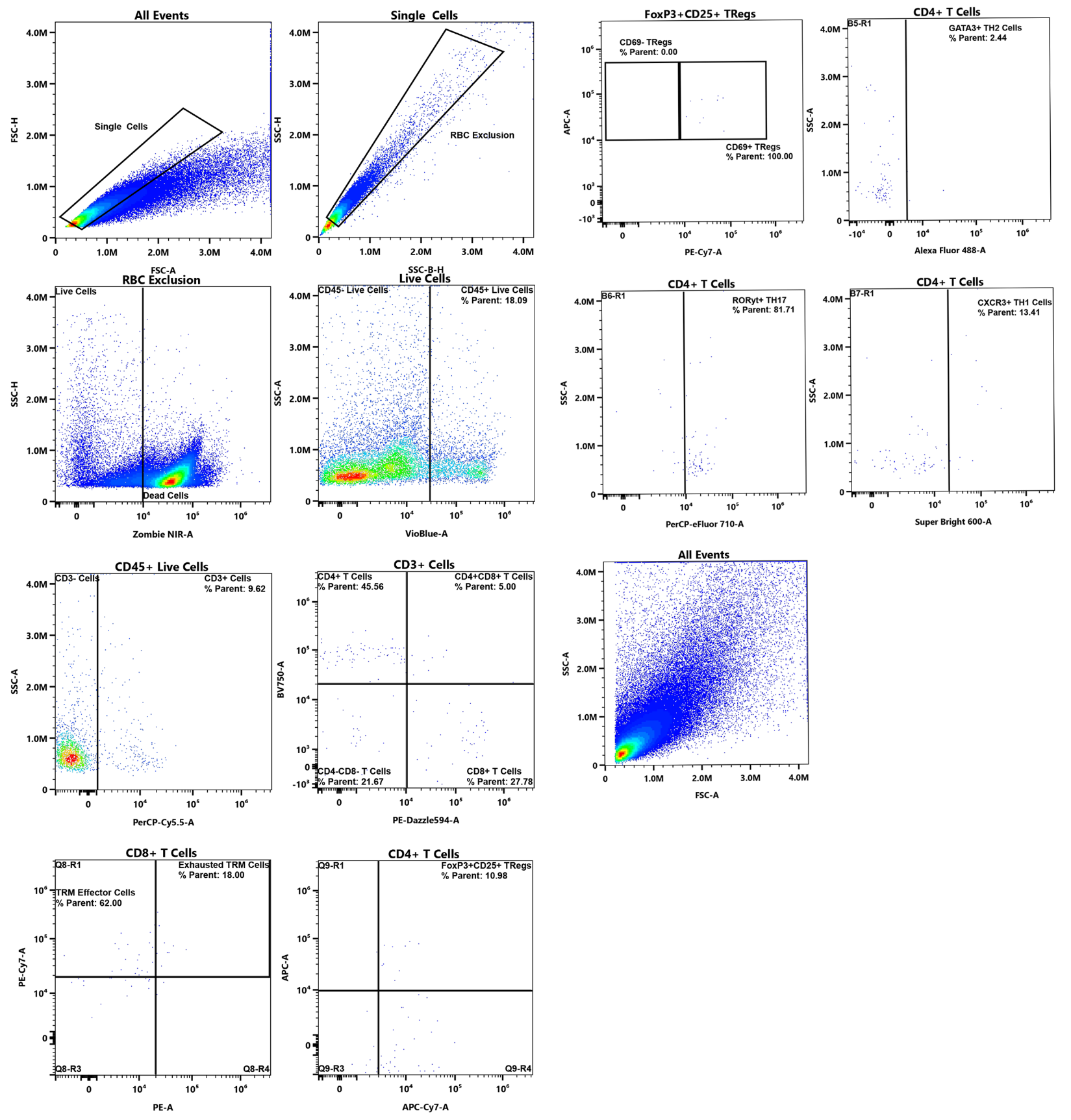


**Supplemental Figure 1.** Representative flow cytometry gating strategy used to identify T cell subpopulations isolated from IL-14αTG and C57BL/6 mouse submandibular glands. Gating was performed using Cytek SpectroFlo software.


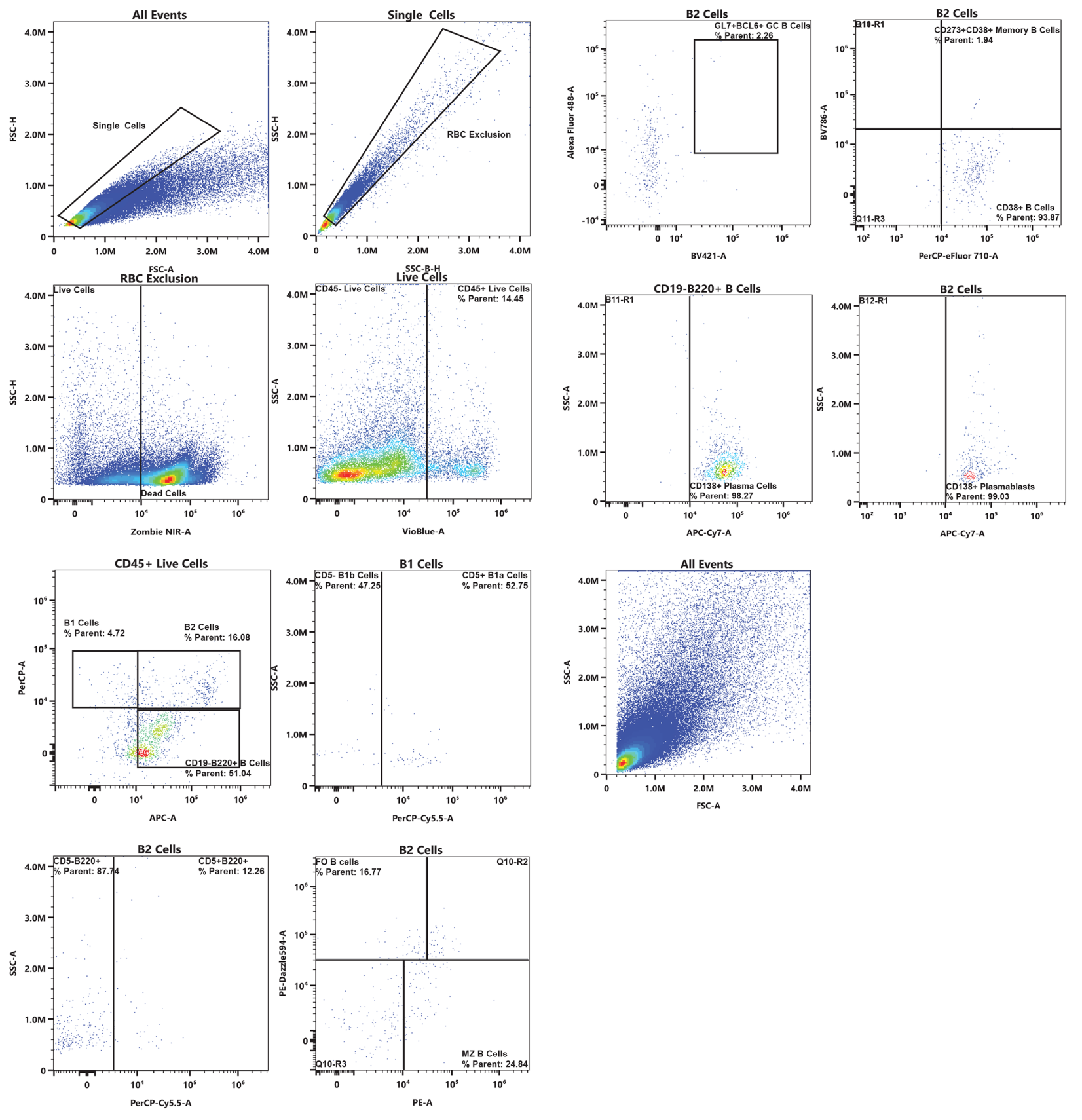


**Supplemental Figure 2.** Representative flow cytometry gating strategy used to identify B cell subpopulations isolated from IL-14αTG and C57BL/6 mouse submandibular glands. Gating was performed using Cytek SpectroFlo software.


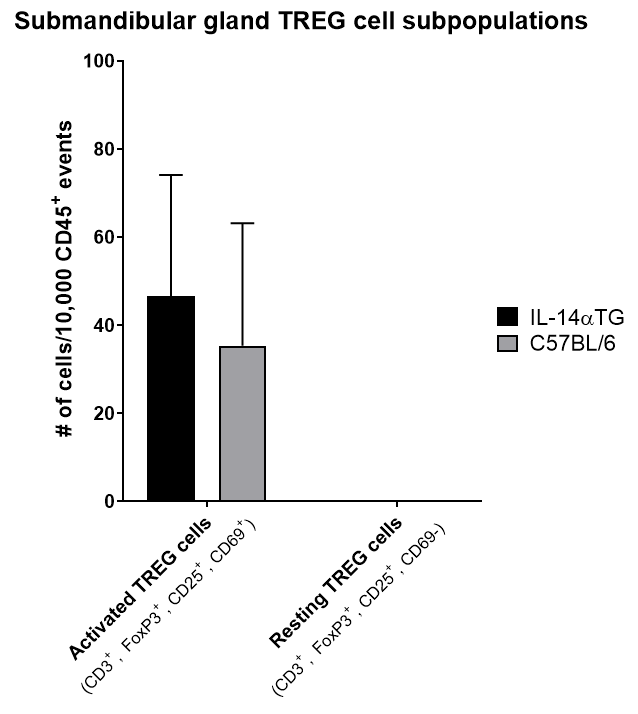


**Supplementary Figure 3.** Activation state of SMG-infiltrating TREG cells based on expression of CD69.


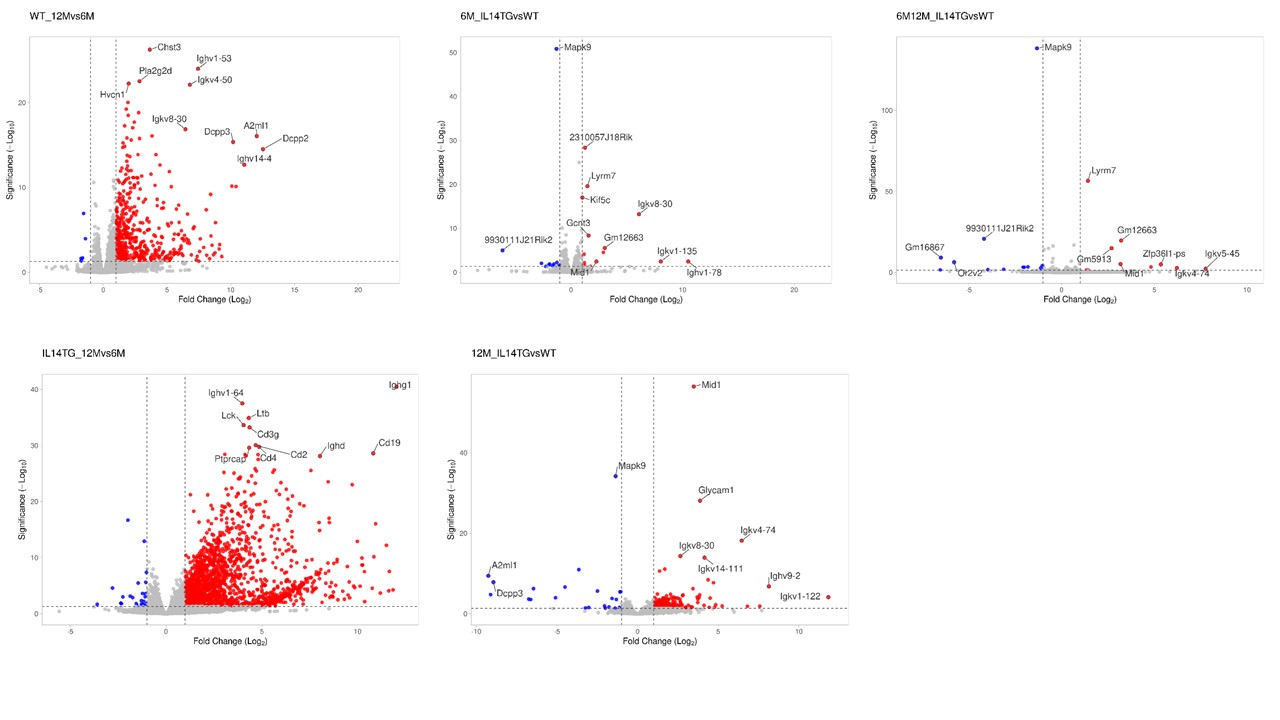


**Supplementary Figure 4.** Volcano plots of differentially expressed genes (DEGs) in submandibular glands from 6-month-old and 12-month-old C57BL/6 and IL-14αTG mice. Annotated dots denote the top 10 DEGs based on Manhattan distance from origin.


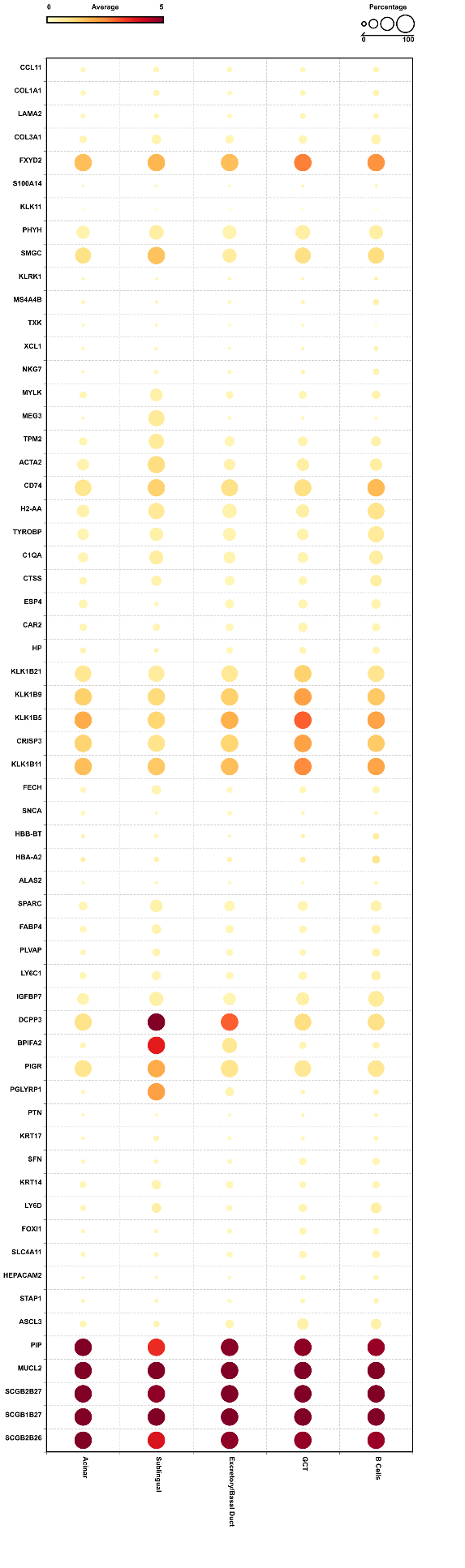

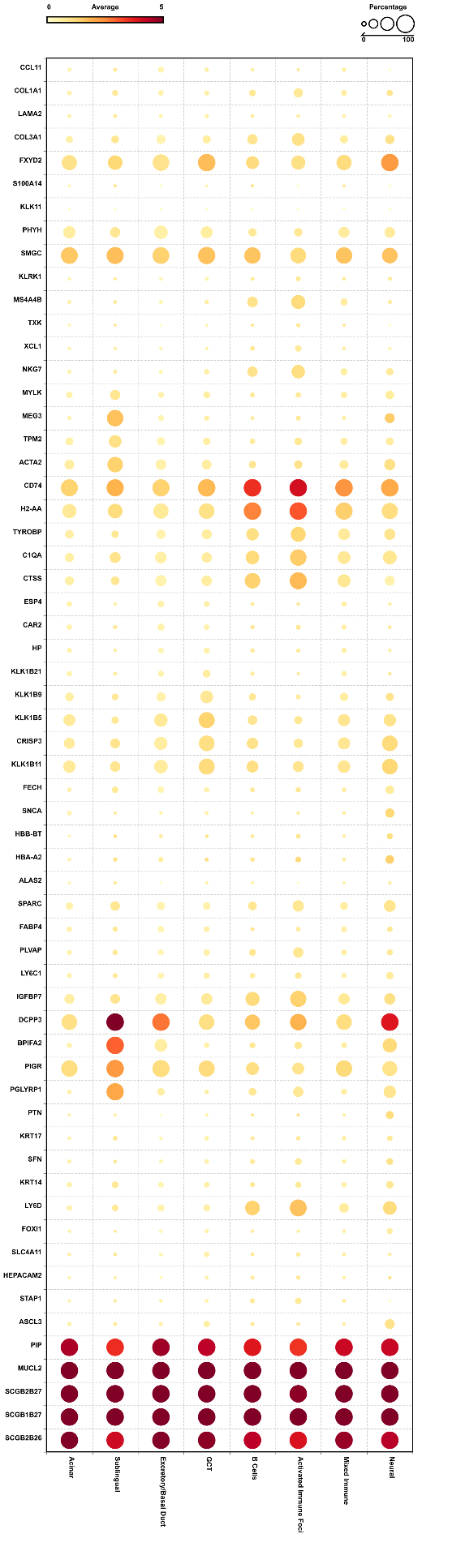
**Supplementary Figure 5.** Dot plot of cell type gene marker expression in 12-month-old (left) IL-14αTG and (right) C57BL/6 mouse salivary gland spatial clusters.


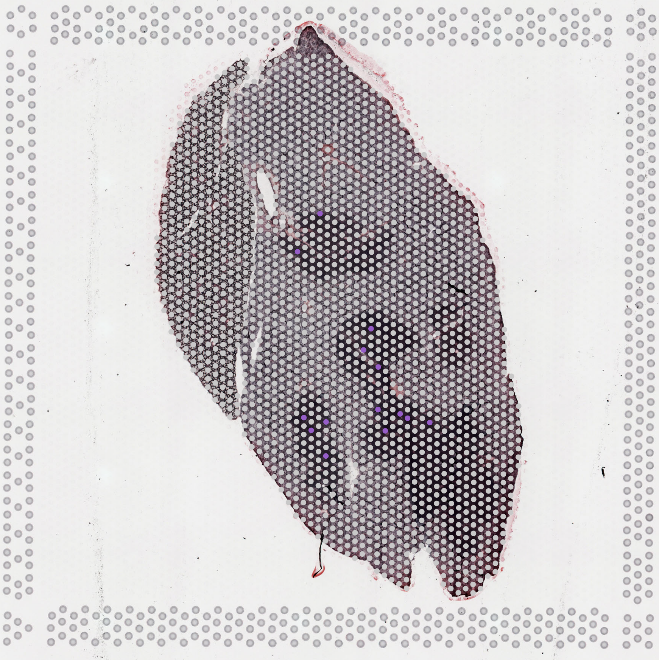

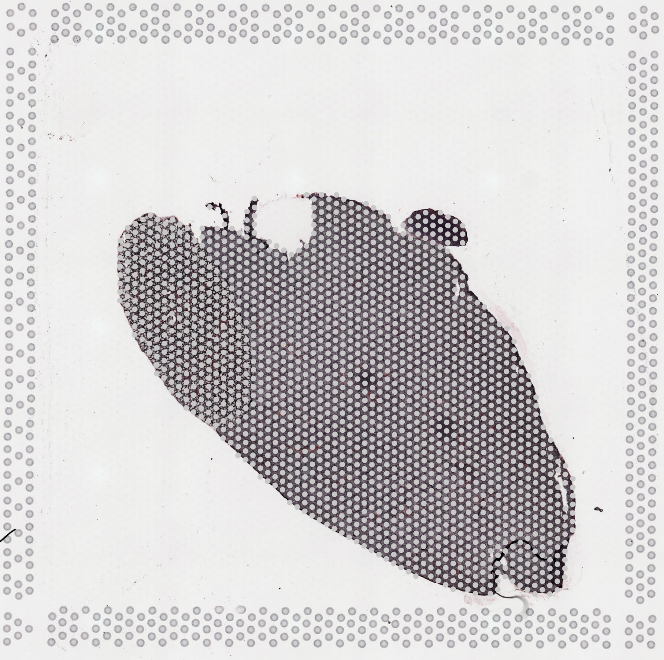


**Supplementary Figure 6.** Barcoded capture areas co-expressing marginal zone B cell markers CD19 and CD21/35 (purple) in SMG from 12-month-old (left) IL-14αTG and (right) C57BL/6 mice.
